# Supplementary material for: Synthetic negative feedback circuits using engineered small RNAs
Source: Nucleic Acids Res. 2018 Sep 13;46(18):9875–89. doi: 10.1093/nar/gky828 (PMC6182179; doi:10.1093/nar/gky828)
Supplement: Supplementary Data [file gky828_supplemental_files.pdf]

Synthetic Negative Feedback Circuits Using Engineered Small RNAs
Supplementary Information

Kelly, C.L., Harris, A.W.K., Steel, H., Hancock, E.J., Heap, J.T., Papachristodoulou, A.

September 1, 2018

S1 Mathematical Modelling

We now outline in detail the mathematical models used for simulation of the systems considered in the main paper. All simulations are performed using MATLAB R2017a. First, in Supplementary Section S1.1 we describe the mathematical structures used to describe regulation at both the transcriptional (e.g. the inducible transcription factors) and translation level (e.g. sRNA inhibition of translation). The complexity of these functional forms represents a trade-off between their ability to capture the behaviour of our systems, and the number of unknown parameters that they depend on. We are therefore dramatically simplifying the description of complex cellular processes, but in doing so we reduce the number of unknown parameters to a level where most can be identified by fitting to experimental data. To achieve this some parameter values must be taken from the literature, as described in Supplementary Section S1.2.

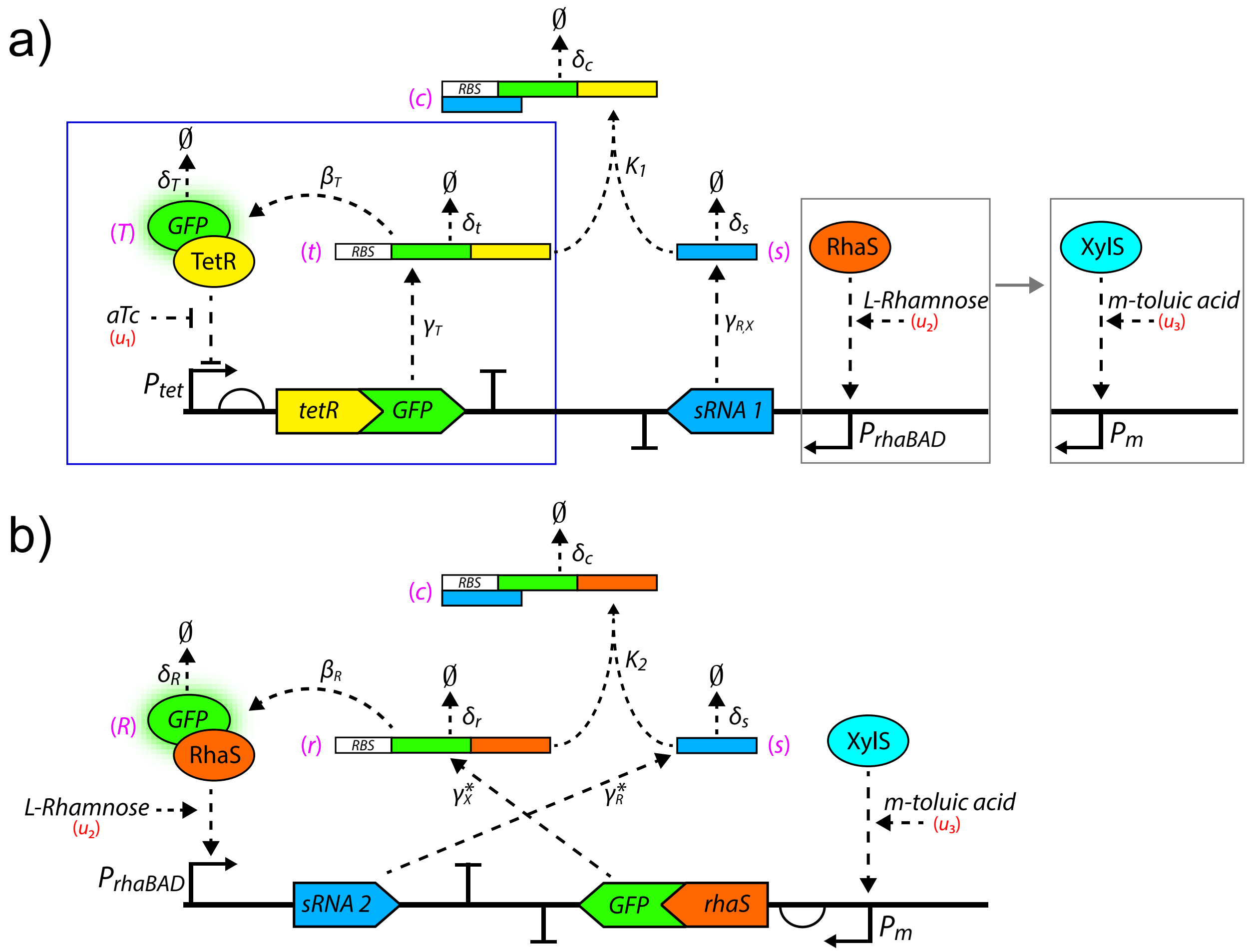

Figure S1: **Architectures for each circuit in this paper.** System components are colour coded across their gene / mRNA / protein forms, and degrade to the null state ∅. Parameters are as defined in the text, state variables considered in modelling are highlighted in purple, and inputs (inducers) are in red. **a)** Layout for the Autorepressor (blue box only), and the sRNA-tuned Autorepressor (whole sub-figure). The alternate XylS inducer system was tested experimentally, which corresponds to exchanging the grey boxes. **b)** Layout for the closed-loop sRNA feedback circuit. The major difference between this and the Autorepressor is apparent: In the latter the sRNA is used to complete a feedback loop, whilst in the former the sRNA is used to tune the effective feedback strength of a standard autorepressing system.

In Sections S1.3-S1.5 we describe the modelling approach for each circuit, for which architectures are outlined in Fig. S1. In each case we provide a simple model, useful for *a priori* analysis of its behaviour, which

is simulated with nominal parameter values. We then describe the individual biochemical reactions taking place, which are expressed as a system of non-linear ordinary differential equations (ODEs). We describe the parameter fitting process for each of these circuits, and compare modelled results to experimental data. We then simulate each circuit's dynamic response to changing inputs, and analyse the influence of intrinsic and extrinsic noise on their behaviour.

S1.1 Model Structures

S1.1.1 The inducible TetR transcription regulation system

We model the transcription rate from P_{tet} as a function of the concentration of the regulatory protein TetR (T) and its inducer anhydroTetracycline (aTc), u_1. This formulation is based upon past studies of this regulatory system [1, 2], and has been shown to accurately model the dynamic and steady-state behaviour of similar systems [3]. The activity of this promoter is given by:

$$\gamma_T = \frac{\alpha_t}{1 + \left(\frac{T}{K_T(1+(u_1/K_{u_1})^{n_{u_1}})}\right)^{n_T}} + \frac{\alpha_L\left(\frac{T}{K_T(1+(u_1/K_{u_1})^{n_{u_1}})}\right)^{n_T}}{1 + \left(\frac{T}{K_T(1+(u_1/K_{u_1})^{n_{u_1}})}\right)^{n_T}} \tag{1}$$

where α_t is the maximum translation rate from the promoter, K_T and K_{u_1} are equilibrium constants that define the system's saturation point in terms of its regulatory protein and inducer respectively, and n_T and n_{u_1} are exponents that define the cooperativity of the TetR and aTc binding processes respectively. Moreover, α_L parameterises the promoter's leakiness, and represents the transcription rate from promoters to which TetR is bound. Alternate approaches to modelling the *tet* promoter exist [4, 5, 6]; we select the form in (1) due to its ability to model autoregulatory systems (as it depends on both TetR and inducer concentration), and the availability of parameters in the literature [3].

S1.1.2 The inducible RhaS transcription regulation system

We model the transcription rate from P_{rhaBAD} as a function of its regulatory protein RhaS (R) and its inducer L-rhamnose (u_2) as follows:

$$\gamma_R^* = \alpha_r^* \frac{\left(\frac{Ru_2}{K_{u_2}^* + u_2}\right)}{K_R + \frac{Ru_2}{K_{u_2}^* + u_2}} \tag{2}$$

where α_r^* parameterises the absolute transcription rate, K_R is an equilibrium constant that defines the system's saturation point in terms of RhaS concentration, and $K_{u_2}^*$ is the cooperativity of the RhaS binding process. The form of (2) mirrors that used to model other activating transcription factors, where a H ill coefficient of 1 is used [3]. This formulation accounts for saturation of the promoter as the concentration of the regulatory protein increases, as well as saturation of regulatory protein activity as inducer increases. For the sRNA-tuned autorepressor (Fig. S1a) the concentration of R is assumed to be constant (constitutive expression), and so in this case (2) simplifies to:

$$\gamma_R = \alpha_r \frac{u_2}{K_{u_2} + u_2} \tag{3}$$

In this case the parameter K_{u_2} depends upon the concentration of regulatory protein R and hence its value is different to that in (2).

S1.1.3 The inducible XylS transcription regulation system

The XylS transcriptional regulatory system is only employed constitutively in our work (that is, we do not regulate the concentration of XylS), and so we model its behaviour using a saturating function of its inducer m-toluic acid (u_3):

$$\gamma_X = \alpha_x \frac{u_3}{K_{u_3} + (u_3)} \tag{4}$$

where α_x parameterises the absolute transcription rate, and K_{u_3} is an equilibrium constant that defines the system's saturation point in terms of its inducer. Here we again have Hill coefficients of 1 for the inducer binding process. For the closed-loop sRNA feedback circuit we replace α_x by α_x^* in (4) to give γ_X^* since the context of P_m, and the sRNA it regulates, changes.

S1.1.4 Translational regulation by sRNA

In the present work we have employed the small-RNA scaffolds described by Na *et al.* [7], which depend upon Hfq chaperone proteins for mediation of sRNA-mRNA binding. Hfq mediation of sRNA-mRNA binding is a complex process for which a range of mathematical models exist [8], with the influence of Hfq varying greatly depending on the particular sRNA employed [9]. Past studies demonstrate that Hfq generally separates quickly from an sRNA-mRNA complex once complementarity is established [10]. This allows it to "actively cycle" between many RNAs targets [11], which facilitates its sharing between natural sRNA regulatory processes and those introduced in synthetic systems. For modelling purposes we have therefore assumed that our synthetic system does not saturate the natural Hfq pool.

To enable parameter fitting, and to simplify our model, we describe Hfq-mediated sRNA-mRNA binding as a first-order reaction of the form:

$$s + t \xrightarrow{K_1} c$$

where (as depicted in Fig. S1) s is sRNA concentration, t is mRNA concentration (r in the case of Circuit 3), and c is the concentration of their bound complex. We assume that the lifetime of complex c is short compared to its un-binding half-life, which justifies modelling of this reaction as uni-directional (i.e. sRNA-mRNA binding is permanent). The approximations we make in this description of the sRNA/mRNA/Hfq interaction will likely result in our model misrepresenting the actual concentration of sRNA, since the active binding and catalysis process mediated by Hfq would provide a higher "apparent" sRNA concentration than is actually present within the cell. This will influence the absolute values of parameters that govern sRNA levels (i.e. its transcription and binding rates), which could quantitatively impact simulations of the system's dynamic response.

S1.2 Parameter selection

The experimental measurements in this study are recorded in terms of fluorescence per cell in arbitrary units. However, because our models describe each species in terms of molecules per cell there is an unknown (ideally linear) relationship between a cell's copy number of GFP, and its total fluorescence. Protein copy numbers for similar circuits typically range from 10^1 to 10^4 per cell [12], with the upper end of this range corresponding to strong expression of stable proteins (such as those considered in our study). Consequently, we assume a one-to-one relation between measured fluorescence and protein copy number. This assumption will mean the value of certain parameters that we fit in our model (for example $\beta_{T,R}$, $K_{1,2}$, and $K_{T,R}$) is inclusive of a fluorescence to copy-number scaling factor, though this will not qualitatively impact the model's behaviour.

Table S1: Parameter values used in modelling each circuit with structures as illustrated in Fig. S1

	Description	Value	Unit	Source
α_t	Transcription rate of *tetR-GFP* gene from P_{tet} in sRNA-tuned Autorepressor	0.85	nM s^{-1}	[13, 14]
α_L	Transcription leak rate of *tetR-GFP* gene from P_{tet} in sRNA-tuned Autorepressor	0.090	nM s^{-1}	Fit in Section S1.4
α_r	Transcription rate of sRNA 1 from P_{rhaBAD} in sRNA-tuned Autorepressor	1.67×10^3	nM s^{-1}	Fit in Section S1.4
α_r^*	Transcription rate of sRNA 2 from P_{rhaBAD} in closed-loop sRNA feedback circuit	1.67×10^3	s^{-1}	Set to α_r
α_x	Transcription rate of sRNA 1 from P_m in sRNA-tuned Autorepressor	841	nM s^{-1}	Fit in Section S1.4
α_x^*	Transcription rate of *rhaS-GFP* gene from P_m in closed-loop sRNA feedback circuit	0.85	nM s^{-1}	[13, 14]
β_T	Translation rate of tetR-GFP mRNA	0.0154	s^{-1}	Fit in Section S1.4
β_T^x	Translation rate of tetR-GFP mRNA (XylS inducer)	0.0224	s^{-1}	Fit in Section S1.4
β_R	Maximum translation rate of rhaS-GFP mRNA	0.0243	s^{-1}	Fit in Section S1.5
K_1	sRNA-mRNA binding rate in sRNA-tuned Autorepressor	7.79×10^{-9}	nM^{-1} s^{-1}	Fit in Section S1.4
K_2	sRNA-mRNA binding rate in closed-loop sRNA feedback circuit	2.24×10^{-7}	nM^{-1} s^{-1}	Fit in Section S1.5
K_T	P_{tet}-TetR binding constant	0.0119	nM	Fit in Section S1.4
K_{u_1}	TetR-aTc binding constant	5.41×10^{-4}	nM	Fit in Section S1.4
K_R	P_{rhaBAD}-rhaS binding constant	1.036×10^6	nM	Fit in Section S1.5
K_{u_2}	RhaS-L-rhamnose binding constant from (3)	2.6×10^3	nM	Fit in Section S1.4
$K_{u_2}^*$	RhaS-L-rhamnose binding constant from (2)	1.4×10^3	nM	Fit in Section S1.5
K_{u_3}	XylS-*m*-toluic acid binding constant	1.78×10^5	nM	Fit in Section S1.5
n_T	P_{tet}-TetR binding cooperativity	3	none	[3]
n_{u_1}	TetR-aTc binding cooperativity	1.11	none	Fit in Section S1.4
δ_t	TetR-GFP mRNA degradation rate	4.1×10^{-3}	s^{-1}	[15]
δ_r	rhaS-GFP mRNA degradation rate	4.1×10^{-3}	s^{-1}	[15]
δ_c	mRNA-sRNA complex degradation rate	4.1×10^{-3}	s^{-1}	[15, 16]
δ_T	TetR-GFP protein degradation rate	3.9×10^{-4}	s^{-1}	[14]
δ_R	RhaS-GFP protein degradation rate	3.9×10^{-4}	s^{-1}	[14]
δ_s	sRNA degradation rate	8×10^{-4}	s^{-1}	[9, 17]

The maximum transcription initiation rates for the *tetR-GFP* and *rhaS-GFP* genes are set as $\alpha = 0.05$ s^{-1} $gene^{-1}$ to correspond to typical levels for a mid-strength promoter in *E. coli* [13, 14]. The plasmids used in our circuits provide mean gene copy numbers of ∼17, which is multiplied by α to give $\alpha_t, \alpha_x^* = 0.85$ s^{-1}. The mRNA degradation rate is set as $\delta_{t,r} = 4.1 \times 10^{-3}$ s^{-1} to represent a typical mRNA half-life of 2.8 minutes for *E. coli* growing in exponential phase [15], and we assume that degradation of the sRNA-mRNA

complex (δ_c) occurs at this same rate (as has been found in sRNA regulatory systems [16]). The protein degradation rate is set as $\delta_{T,R} = 3.9 \times 10^{-4}\ s^{-1}$ to correspond to dilution during growth with a 30 minute doubling time [14]. For the sRNA we sum its dilution rate (equal to $\delta_{T,R}$) with its measured degradation rate of $\delta_{deg} = 4.1 \times 10^{-4}\ s^{-1}$ [17] (which is an order of magnitude slower than typical mRNA due to the stabilising effect of Hfq [9]) to give $\delta_{m_s} = 8 \times 10^{-4}\ s^{-1}$. The precise values of degradation rates are not critical for the fitting of our models to steady-state circuit outputs: Each biochemical reaction is a trade-off between creation and degradation, and thus a changed value of δ can be countered by a change in creation rate (to yield an equivalent equilibrium species abundance). However, the dynamic behaviour of our system *is* a function of these assumed parameters, and hence any quantitative conclusions (e.g. estimations of response time) drawn from dynamic simulations will depend upon their accuracy.

For the model of activity for the *tet* promoter (1) we set $n_T = 3$, as found in past studies of similar autorepressor systems [3]. Cooperativity of aTc binding to TetR (n_{u_1}) was fit to the experimental data, as there was a large degree of uncertainty in its reported value [3]. To convert inducer units (typically ng/ml) into concentrations we use molecular weights of 426 g mol^{-1} for aTc, 164 g mol^{-1} for L-rhamnose, and 136 g mol^{-1} for *m*-toluic acid. All other parameters described in Fig. S1 and Supplementary Section S1.1 were fit to experimental data as discussed in subsequent sections. Assuming a cell volume of 0.6 μm^3 a 1 nM concentration corresponds to approximately 1 molecule per cell, and thus parameters value (summarised in Table S1) can easily be converted to units of molecules per cell for use in stochastic simulations.

S1.3 The Autorepressor

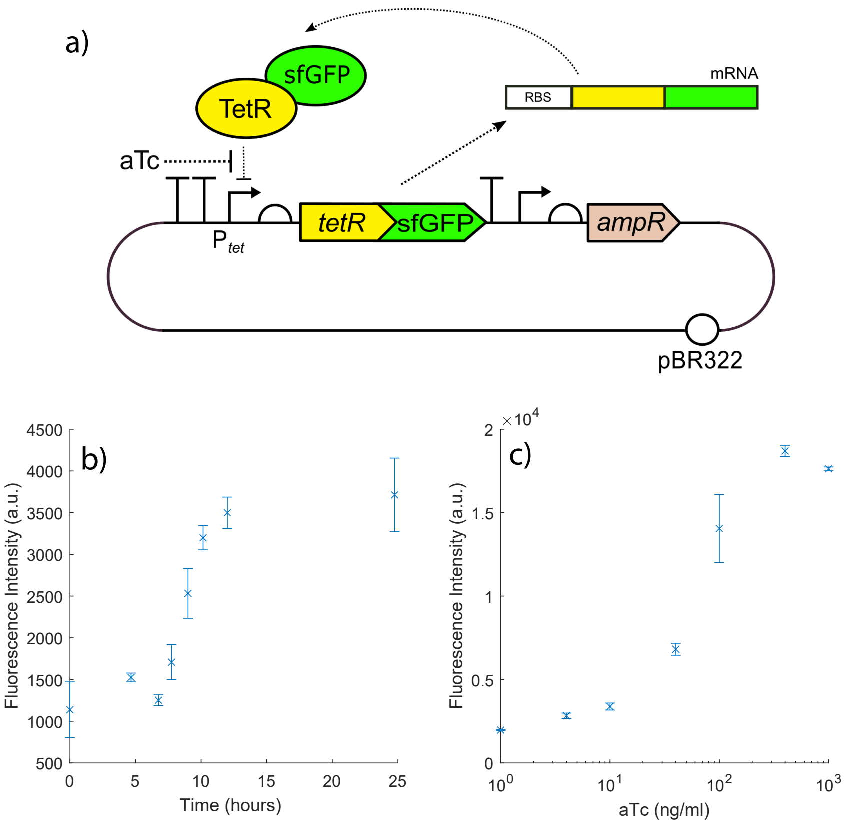

Figure S2: **Schematic and experimental validation of the autorepressor-encoding plasmid pCK200.** **a)** The autorepressor plasmid pCK200 was designed and constructed, consisting of a fusion of the genes encoding the tet repressor (TetR) and superfolder GFP (sfGFP) placed immediately downstream of the tetracycline-responsive P_{tet} promoter and a strong synthetic RBS. **b)** Timecourse of TetR-sfGFP autoregulation. *E. coli* MG1655 cells containing pCK200 were cultured in minimal M9 media at 37 °C and GFP fluorescence measured by flow cytometry at the specific timepoints shown. **c)** Input-output response of the autorepressor circuit to aTc. *E. coli* MG1655 cells containing pCK200 were cultured at 37 °C in EZ rich defined medium with specified concentrations of aTc and the GFP fluorescence measured at late-exponential phase (5 h) by flow cytometry. Fluorescence intensity represents the geometric mean of fluorescence. Error bars shown represent the standard deviation of three independent biological replicates.

S1.3.1 Simple Model

In order to allow *a priori* investigation of our system's behaviour we build a simple model of the autorepressor, of the form:

$$\frac{dX}{dt} = \beta L + \frac{\beta(1-L)}{1+\left(\frac{X}{0.01+u_1}\right)^3} - X \tag{5}$$

where X is the system's protein concentration, L is a leakage term, and β is a noise term that will be used to introduce variabily. This model reflects those used previously to describe the autorepressor [3], though it is largely de-parameterised as we only intend to investigate its qualitative behaviour. We calculated the steady-state value of X as u_1 is varied in Fig. 1a of the main manuscript (simulation where $u_2 = 0$), finding good qualitative agreement with our experimental observations of the autorepressor's behaviour (Fig. S2, Fig. S3). We crudely simulate the influence of extrinsic noise on this system by sampling the parameter β

from a log-normal distribution (for detailed discussion see Section S1.4.5) with a mean of one, finding that the output achieves minimal variability for intermediate induction levels (as observed experimentally in past studies [12]).

S1.3.2 Detailed Model

We construct a more detailed model for the Autorepressor (Fig. S1a - blue box) that depends on two states: The concentration of mRNA (t) and protein (T) for the TetR-GFP complex. The underlying biochemical reactions can be expressed as:

$$\emptyset \xrightarrow{\gamma_T} \mathrm{t} \xrightarrow{\delta_t} \emptyset,$$

$$\mathrm{t} \xrightarrow{\beta_T} \mathrm{t} + \mathrm{T},$$

$$\mathrm{T} \xrightarrow{\delta_T} \emptyset,$$

where γ_T is defined as in (1), and β_T is the maximal translation rate (Fig. S1a). These reactions can be described using two coupled differential equations of the form:

$$\begin{aligned} \dot{t} &= \gamma_T - \delta_t t \\ \dot{T} &= \beta_T t - \delta_T T \end{aligned} \qquad (6)$$

S1.3.3 Parameter Fitting

We initially set $\alpha_L = 0$ in γ_T and use least-squares minimisation to fit the unknown parameters K_{u_1}, K_T, n_{u_1}, and β_T such that the steady-state output of the model (6) best matches the experimental data. For this we use the measurements of the sRNA-tuned Autorepressor (pCK221) with the *rhaBAD* promoter when sRNA is not induced (concentration of L-rhamnose is zero). Experimental data and the model fit are presented in Fig. S3a. We observe that the model accurately captures the system's behaviour over the range of inducer concentrations measured, and that it correctly approximates the low- and high-inducer saturation levels anticipated from this promoter.

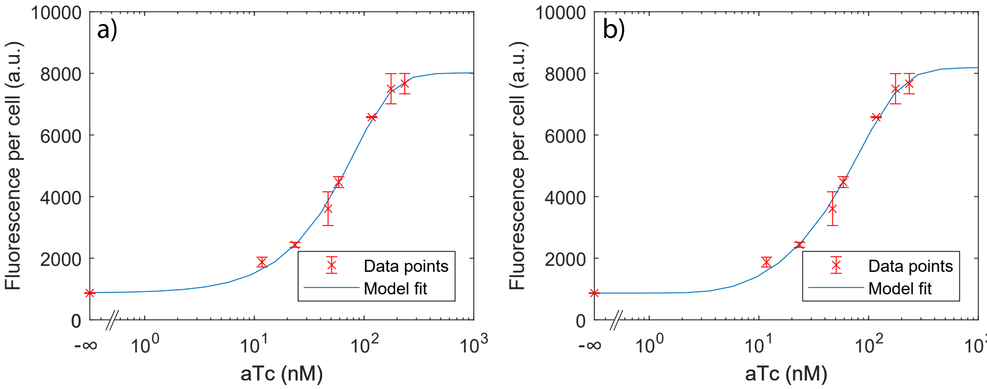

Figure S3: **Autorepressor data and model fit.** **a)** Model fit with $\alpha_L = 0$, yielding $K_{u_1} = 8.98$ nM, $K_T = 433$ nM, $n_{u_1} = 1.23$, and $\beta_T = 0.0151$ s^{-1}. Data points are measurements of the sRNA-tuned Autorepressor (pCK221) with the RhaS induction system but zero L-rhamnose. **b)** Model fit from Section S1.4, yielding $K_{u_1} = 5.41 \times 10^{-4}$ nM, $K_T = 0.0119$ nM, $n_{u_1} = 1.11$, $\beta_T = 0.0154$ s^{-1}, and $\alpha_L = 0.090$ nM s^{-1}. These parameters are those summarised in Table S1. Error bars shown represent the standard deviation of three independent biological replicates.

From this data set alone it is not possible to uniquely identify the parameters K_{u_1}, K_T, n_{u_1}, and β_T in addition to α_L. This is because there are two possible contributing factors to the $u_1 \to 0$ limit of the promoter's output (1): First, if all promoters are bound to TetR at all times, then the $u_1 = 0$ output level is determined by α_L (this is considered in [1]). However, it is also possible for there to be a non-zero output

when $u_1 = 0$ in the absence of leakage ($\alpha_L = 0$) if some promoters are not bound to TetR, as determined by the value of K_T. It is not possible to distinguish the relative contribution of these two mechanisms from experiments in which only aTc concentration is varied, and thus we must fit all parameters for this circuit simultaneously in Section S1.4, in which the apparent value of α_t is varied due to sRNA induction. The result from this fit is presented in Fig. S3b, demonstrating close correspondance to Fig. S3a. When $\alpha_L \neq 0$, K_{u_1} takes a much smaller value, as this parameter is no longer required in determining the system's output level when $u_1 = 0$. Consequently, given the present data the absolute values of K_{u_1} and K_T are only weakly identifiable. However, the quantity $(K_T)^{1/n_{u_1}}/K_{u_1}$ which arises in (1) for high inducer concentrations ($u_1 > K_{u_1}$) *is*: It is equal to 15.50 when $\alpha_L = 0$ and 34.12 when $\alpha_L = 0.09$, and thus the system's sensitivity to TetR concentration (T) is similar in both cases.

S1.4 sRNA-Tuned Autorepressor

S1.4.1 Simple Model

To investigate the anticipated behaviour of this circuit prior to experimental implementation we extend the simple model of the autorepressor proposed in Section S1.3.1 to include the interaction of sRNA:

$$\frac{dX}{dt} = \beta L + \frac{\beta(1-L)}{1+\left(\frac{X}{0.01+u_1}\right)^3} - X - u_2 X \tag{7}$$

Here the final term ($u_2 X$) approximates the contribution of the inhibitory sRNA, which acts proportionally to the quantity of its targets (the mRNA that would be transcribed to produce X) and its induction level u_2. We calculated the steady-state value of X as u_1 is varied in Fig. 1a of the main manuscript (for a range of sRNA levels determined by u_1), demonstrating the sRNA's ability to tune the system's response to u_1 (aTc).

S1.4.2 Detailed Model

We now extend the autorepressor model considered in Section S1.3 to include two additional states that account for sRNA inhibition of translation: The concentration of sRNA (s), and the concentration of the sRNA-mRNA complex (c). The underlying biochemical reactions can be expressed as:

$$\begin{aligned}
\emptyset &\xrightarrow{\gamma_T} \mathrm{t} \xrightarrow{\delta_t} \emptyset,\\
\emptyset &\xrightarrow{\gamma_{R,X}} \mathrm{s} \xrightarrow{\delta_s} \emptyset,\\
\mathrm{t}+\mathrm{s} &\xrightarrow{K_1} \mathrm{c} \xrightarrow{\delta_c} \emptyset,\\
\mathrm{t} &\xrightarrow{\beta_T} \mathrm{t}+\mathrm{T},\\
\mathrm{T} &\xrightarrow{\delta_T} \emptyset,
\end{aligned}$$

where γ_R and γ_X are defined for the different inducer systems as in (3) and (4) respectively, K_1 is the binding rate of sRNA and mRNA, and β_{L_1} is the translation leak rate from the sRNA-mRNA complex. These reactions can be described using four coupled differential equations of the form:

$$\begin{aligned}
\dot{t} &= \gamma_T - \delta_t t - K_1 ts\\
\dot{s} &= \gamma_{R,X} - \delta_s s - K_1 ts\\
\dot{c} &= K_1 ts - \delta_c c\\
\dot{T} &= \beta_T t - \delta_T T
\end{aligned} \tag{8}$$

where the choice of γ_R or γ_X depends on the inducible promoter used (either P_{rhaBAD} or P_m) to control sRNA expression.

S1.4.3 Parameter Fitting

We initially set $\alpha_L = 0$ and use least-squares minimisation to fit the unknown parameters K_1, K_{u_2}, and α_r in the model (8) such that its steady-state behaviour best matches the data presented in Fig. 3 (main text). Here we are using the parameters fit for the zero L-rhamnose data in Fig. S3a when $\alpha_L = 0$. Experimental data and the model fit are presented in Fig. S4a. We observe that the model replicates the data well when aTc$\geq$ 5 ng ml^{-1}, but not so well as L-rhamnose is increased in the zero aTc case. This discrepancy is a fundamental property of our model when $\alpha_L = 0$: It is impossible for the fold-change of output due to sRNA induction (that is, the output when L-rhamnose equals zero divided by the output when L-rhamnose is 0.001 ng/ml) to be greater at low aTc concentrations than at high aTc concentrations. Due to this phenomenon we conclude that when no aTc is present the system's output level is determined to a substantial degree by leakage from TetR-bound promoters (discussed in Section S1.1), thus requiring $\alpha_L \neq 0$.

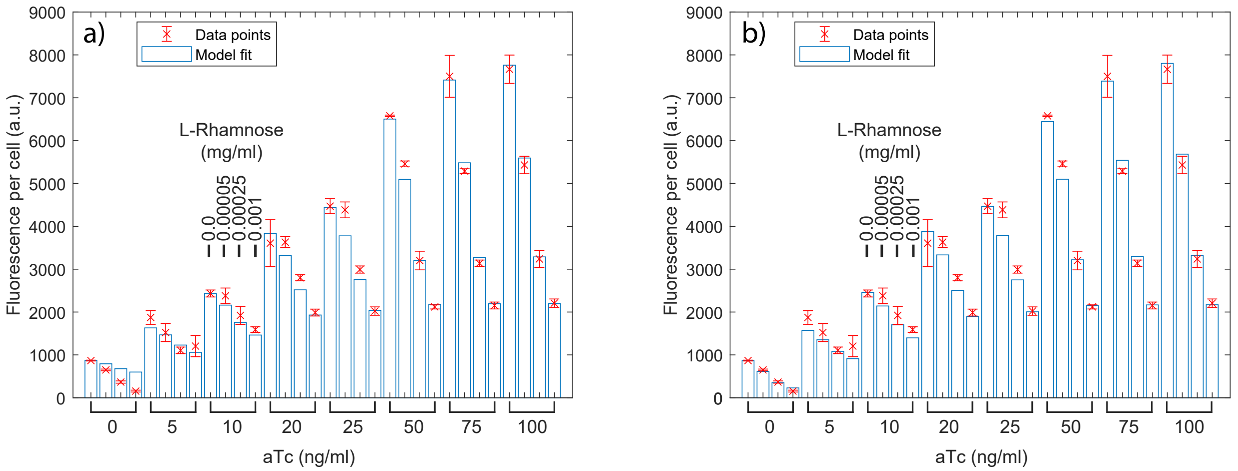

Figure S4: **sRNA-tuned Autorepressor with RhaS, data and model fit. a)** Model fit for circuit in Fig. S1a with RhaS induction system. In this case we set $\alpha_L = 0$ and fit the three remaining parameters, yielding $K_1 = 1.080 \times 10^{-8}$ nM^{-1}, $K_{u_2} = 2.4 \times 10^3$ nM, and $\alpha_r = 1.12 \times 10^3$ s^{-1}. Other parameters are as in Fig. S3a. We observe good agreement between model and experiment when aTc ≥ 5 ng ml^{-1}, but when aTc $= 0$ there is substantial discrepancy. **b)** Model fit with α_L allowed to vary. Resulting parameter values are as in Table S1. We now find good agreement between model and data across all input combinations. Error bars shown represent the standard deviation of three independent biological replicates.

We now allow α_L to vary and fit all free parameters (K_{u_1}, K_{u_2}, K_T, n_{u_1}, β_T, α_L, K_1, and α_r), recognising that there will be a large amount of slack in the relative values of K_T and K_{u_1} as discussed in Section S1.3. The resulting parameters are listed in Table S1, and we observe good agreement (Fig. S4b) across all inducer concentrations, with the model accurately capturing the system's behaviour for 0 aTc. Without aTc induction TetR concentration is predominantly a function of promoter leakiness, which means that a reduction in the concentration of T has little impact upon translation from P_{tet}. Therefore, the sRNA is able to achieve its maximal repression fold with the L-rhamnose inducer saturated, reducing the system's output to ~ 0.2 times its value when L-rhamnose is not present. This is not the case when aTc ≥ 5, since the dependence of (1) on both u_1 and T means that decreased translation of tetR mRNA due to sRNA-mRNA binding results in higher promoter activity, which feeds back to provide more free mRNA.

We now fit our models to the second variant of the sRNA-tuned Autorepressor, in which the RhaS induction system is replaced by XylS (pCK226, Fig. S5a). Since the autorepressor circuit and sRNA interaction remain unchanged we maintain most parameter values found for the RhaS circuit variant. We only fit β_T^x and α_x, and take the value of K_{u_3} found in Section S1.5. We first fit our model in the absence of m-toluic acid (Fig. S5b), yielding $\beta_T^x = 0.0224$ s^{-1}. Theoretically this value should be identical to that found in the RhaS version of Circuit 2 ($\beta_T = 0.0154$ s^{-1}): In neither case is the sRNA component of this circuit induced, so assuming minimal promoter leakage translation of identical mRNA should occur at the same rate. We hypothesise that their $\sim 30\%$ difference is likely due to the anticipated variability in inocculants between different experiments, as well as any differing burden effects that arise due to the constitutive expression of RhaS vs XylS within the cell. We then fit the value of α_x to the whole experimental data set, and we observe that the model is able to capture our system's behaviour (Fig. S5c), including at low aTc levels.

S1.4.4 Dynamic Behaviour

To assess the dynamic behaviour of our feedback system we numerically integrate the system of equations (8) and apply a dynamic input to each inducer in turn. In doing so we are implicitly assuming that some reactions within our circuit (for example, inducer-transcription factor binding) occur quickly, since we do not explicitly model these. This assumption has been made in past studies which investigated the dynamic

behaviour of similar transcription-factor systems [3] and was found to correspond well with experimental results. Other factors that introduce a time-delay, such as the uptake of inducer molecules into cells, are also not considered, and so simulated response times will under-estimate those achievable experimentally. We introduce a dynamic signal to our system's inducers by setting:

$$u_i(t) = u_i^*(t) \times u_i^0 \tag{9}$$

where the time-dependent profile u_i^* determines the magnitude of variation for the inducer input i about its baseline value u_i^0. Results for these simulations for the sRNA-tuned Autorepressor with the RhaS induction system are presented Fig. S6. We observe that it is possible to reduce the system output's sensitivity to one input, whilst maintaining a given mean output, by using the other input as a tuning dial. For example, in Fig. S6 if $u_1^0 = 20$ ng ml^{-1} and $u_2 = 0$ ng ml^{-1} we see high sensitivity to fluctuations in u_1. However, if we set $u_1^0 = 75$ ng ml^{-1} and $u_2 = 1.45 \times 10^{-4}$ ng ml^{-1} the system's mean output remains the same, but equal percentage-change variations in u_1 have a substantially reduced impact. This is due to the sRNA (controlled by u_2) shifting the autorepressor's negative feedback equilibrium further towards saturation, reducing its sensitivity to changes in u_1.

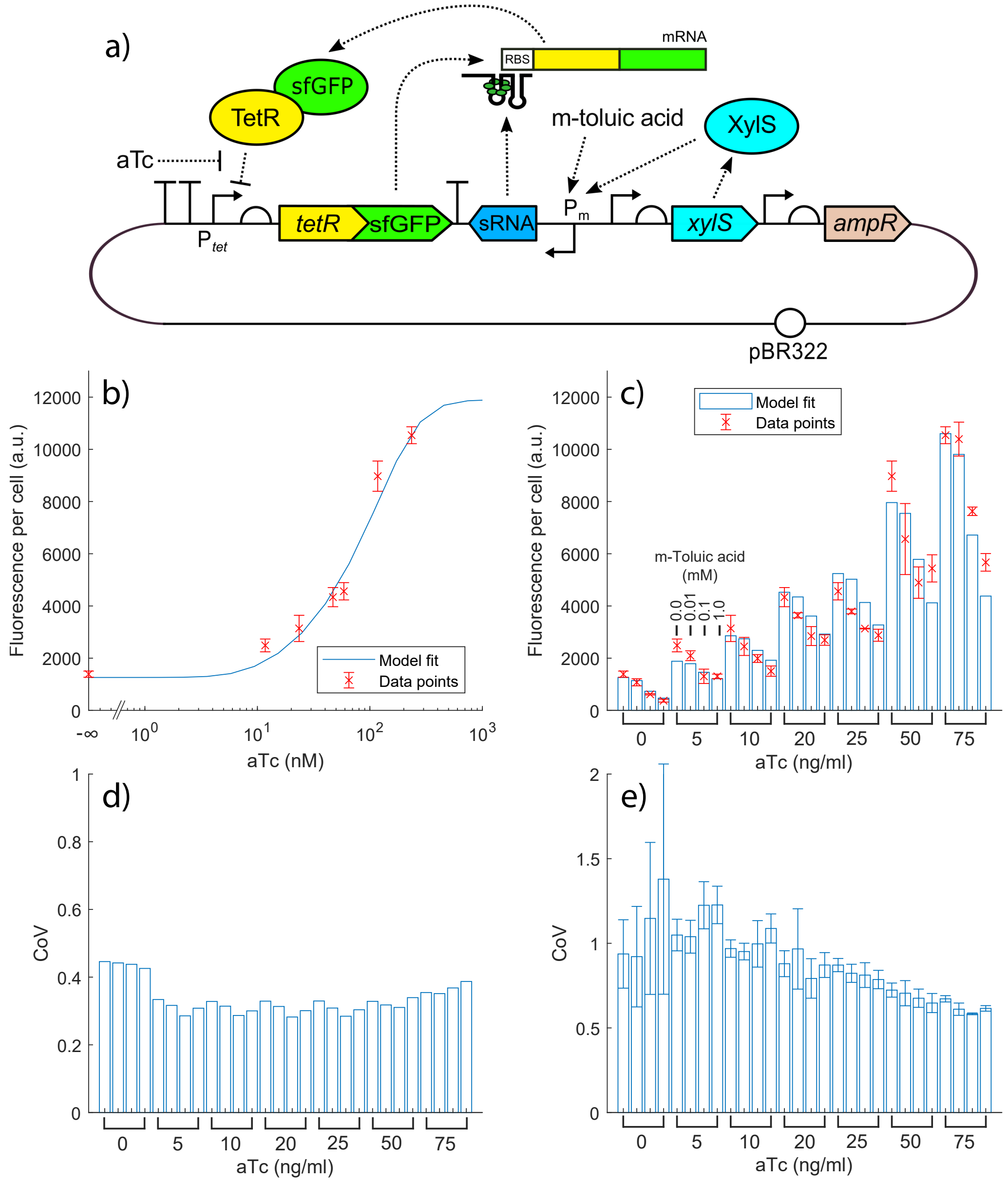

Figure S5: **sRNA-tuned Autorepressor with XylS, data and model fit.** **a)** Schematic diagram of plasmid pCK226. **b)** Validation of the autorepressor loop with increasing concentrations of aTc. *E. coli* strain JW3876 was transformed with pCK226, cultured at 37 °C in EZ rich defined medium supplemented with glycerol and increasing concentrations of aTc and GFP fluorescence measured at late exponential phase (5 h) by flow cytometry (red X). Predicted input-output response was simulated and is overlayed to show model fit (blue line). **c)** Testing the ability to fine-tune the coarse aTc input-output dial with the inducible expression of the sRNA. *E. coli* strain JW3876 was transformed with pCK226, cultured at 37 °C in EZ rich defined medium supplemented with glycerol and increasing concentrations of *m*-toluic acid and aTc and GFP fluorescence measured at late exponential phase (5 h) by flow cytometry (blue columns). Simulated steady-state output is overlayed to show model fit (red X). **d)** Predicted coefficients of variation in the TetR-sfGFP mean when output is tuned via both external inputs. Simulations include the influence of both extrinsic and intrinsic noise as described in Section S1.4.5. **e)** Experimentally-obtained coefficients of variation around the TetR-sfGFP mean when output is tuned using both *m*-toluic acid and aTc. Fluorescence intensity represents the geometric mean of fluorescence. Error bars shown represent the standard deviation of three independent biological replicates.

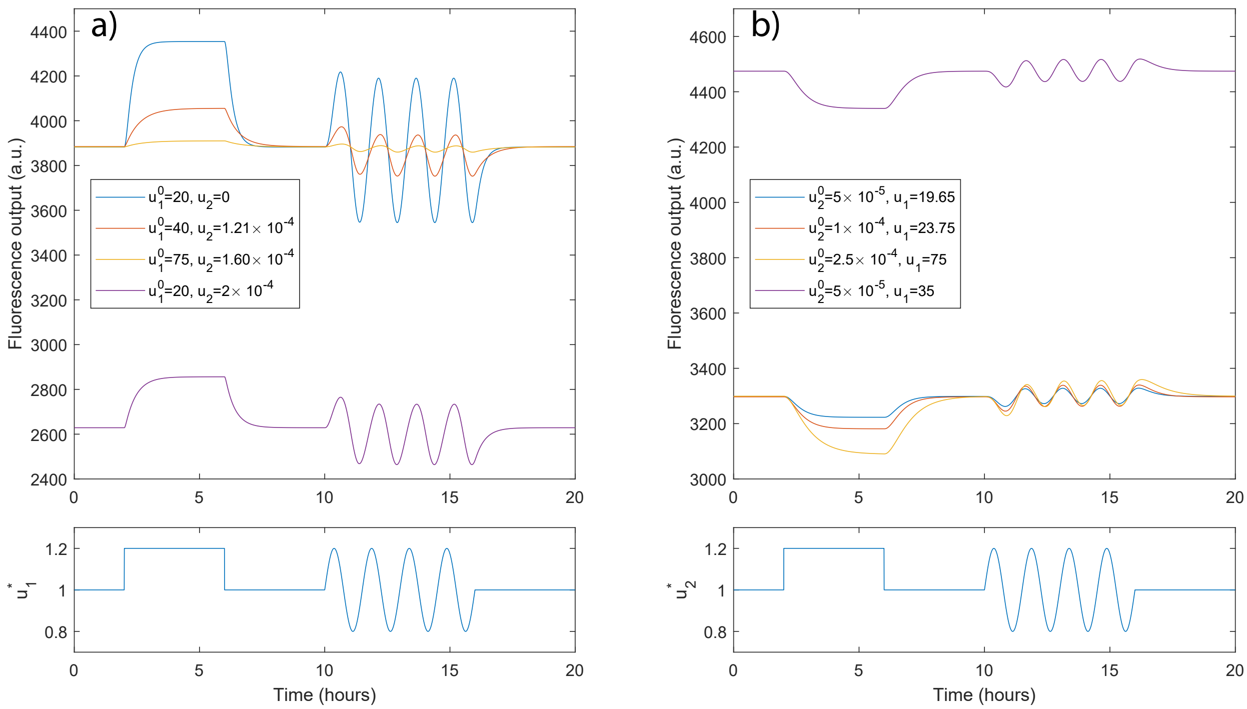

Figure S6: **Dynamic simulations of the sRNA-tuned Autorepressor.** All inducer concentrations are in ng ml^{-1}. **a)** System output over time as u_1 (aTc) is varied according to (9) with the u_1^* profile illustrated. **b)** Similar to (a) but with u_2 (L-rhamnose) varying over time. We observe a slower response to changes in u_2, and smaller output fluctuations for a given fold-change in this inducer.

S1.4.5 Noise Simulation

To assess the impact of variability on our system we simulate its behaviour in the presence of intrinsic (due to stochasticity of the system's biochemical processes) and extrinsic (due to fluctuations in the concentration and state of cellular components that it interacts with) noise types [18, 19]. Extrinsic noise includes both short- and long-term phenomena: A circuit's behaviour can be impacted by temporary fluctuations in cellular machinery abundance [20] (such as as ribosome sequestration, which impacts translation rate in synthetic circuits [21, 22]), or epigenetic differences in proteome and cell state that are passed on as cells divide [23, 24, 25]. Uneven distribution of proteins during cell division [26] can similarly introduce extrinsic fluctuations, as can environmental variability between cells (for example, temperature heterogeneities). Total gene expression noise is thus a function of both cell-wide fluctuations, as well as variability in gene-specific regulation [27]. In general it can be challenging to identify the contribution that each noise type has to a system's total noise level [28] due to the interplay between different sources [29], though for many synthetic systems extrinsic noise is the greater contributor [18, 29, 30, 31]. We therefore proceed by modelling different noise types independently, and then demonstrate the anticipated outcome when combined.

In the following we quantify noise in terms of the Coefficient of Variation (CoV) of the output (concentration of protein T) in a population, which is a metric commonly used in studies of gene expression noise [32]. For log-normally distributed data (as is often found in biological systems [33, 34]) CoV is given by:

$$CoV(T) = \sqrt{e^{s_{ln}^2} - 1} \tag{10}$$

where $s_{ln} = \sigma(ln(T))$ is the standard deviation of the natural logarithm of the variable T.

First we simulate intrinsic variability of the system in (8) with parameters as in Table. S1 using the tau-leaping method, based on the Gillespie algorithm [5]. Such an approach treats each biochemical reaction

as a stochastic process, probabilistically incrementing each species abundance by an integer amount in each time step depending upon the calculated reaction rate. To account for stochastic variability in plasmid copy number we introduced a new equation to describe plasmid dynamics:

$$\dot{P} = \delta_P(P_0 - P) \tag{11}$$

where we set the plasmid degradation rate $\delta_P = \delta_T$ to correspond to dilution by cell growth, and P_0 is the mean plasmid concentration, which we took to be 17. The RNA transcription terms in (8)a,b were then scaled by the factor $P(t)/P_0$ to account for variation in transcription due to stochastic variation in plasmid copy number.

It is likely that stochastic simulation of (8) will under-estimate noise intrinsic to our circuit's peformance, since this system of equations lumps many complex processes into single parameters, and thus the influence of stochasticity at these steps is not modelled. For example, the Hfq-mediated binding of sRNA/mRNA is described by the single parameter K, which does not account for any stochasticity in intermediate steps (which occur at low species concentrations, where the influence of biochemical stochasticity is greatest [5]). We run stochastic simulations of a population of 10^5 individual cells until the population mean concentration of each species reaches an equilibrium, and then calculate the mean CoV of the equilibrated population over a further 1 hour of simulation. Results are presented in Fig. S7 a, demonstrating that the magnitude of intrinsic noise is generally low ($CoV < 0.2$), and that for a given aTc (u_1) concentration noise decreases as sRNA concentration is increased (i.e. u_2 (L-rhamnose) increasing).

We model the impact of extrinsic noise in our system by sampling individual parameter values from a log-normal distribution, and then observing the spread of output levels in a cell population. In reality extrinsic noise sources would have some impact upon every parameter's value, but we must select a few likely places for introduction of noise to make our simulations tractable. The particular parameters chosen, and the magnitude of the variability introduced at each, are ultimately somewhat arbitrary, and we will therefore be more interested in qualitative trends in our simulation results rather than the absolute levels predicted. This considered, we elect to introduce noise in gene expression at the translational step, and in the uptake/mixing of each inducer, by setting:

$$\beta_T^* = \Delta_1 \times \beta_T,\ u_{1,2}^* = \Delta_2 \times u_{1,2} \tag{12}$$

where β_T^* and $u_{1,2}^*$ are the translation rate and inducer concentration parameters with noise introduced, and $\Delta_{1,2} \sim$ log-normal(μ, σ^2) are independent random parameters sampled from a log-normal distribution for which we set $\mu = -\sigma^2/2$ such that the distribution's mean is unity. For the system in (8) we simulate a population of 10^5 cells with parameters sampled as described with $\sigma = 0.4$, for which results are presented in Fig. S7b. We observe qualitatively similar behaviour to that predicted by our simple model of this system (Fig. 1 of the main text): a reduced level of variability is found for intermediate levels of aTc (u_1). The magnitude of variation introduced due to extrinsic noise here is substantially greater than that of intrinsic noise, though this comparison ultimately depends on the value chosen for σ.

In a real biological system it is difficult to separate measurements of noise introduced by intrinsic and extrinsic factors due to the influence of each factor depending on that of the other [28]. To investigate how the interplay of these sources would impact our model system we perform a combined simulation of both sources: we stochastically simulate our system (introducing intrinsic fluctuations) with parameters varied according to log-normal distributions as described above (to introduce extrinsic sources). Results from this simulation are presented in Fig. S7c, and are similar that observed for simulations of extrinsic noise alone (Fig. S7b). Qualitatively, the predicted trends as aTc and L-rhamnose concentration are varied match well with those measured experimentally (Fig. S7d).

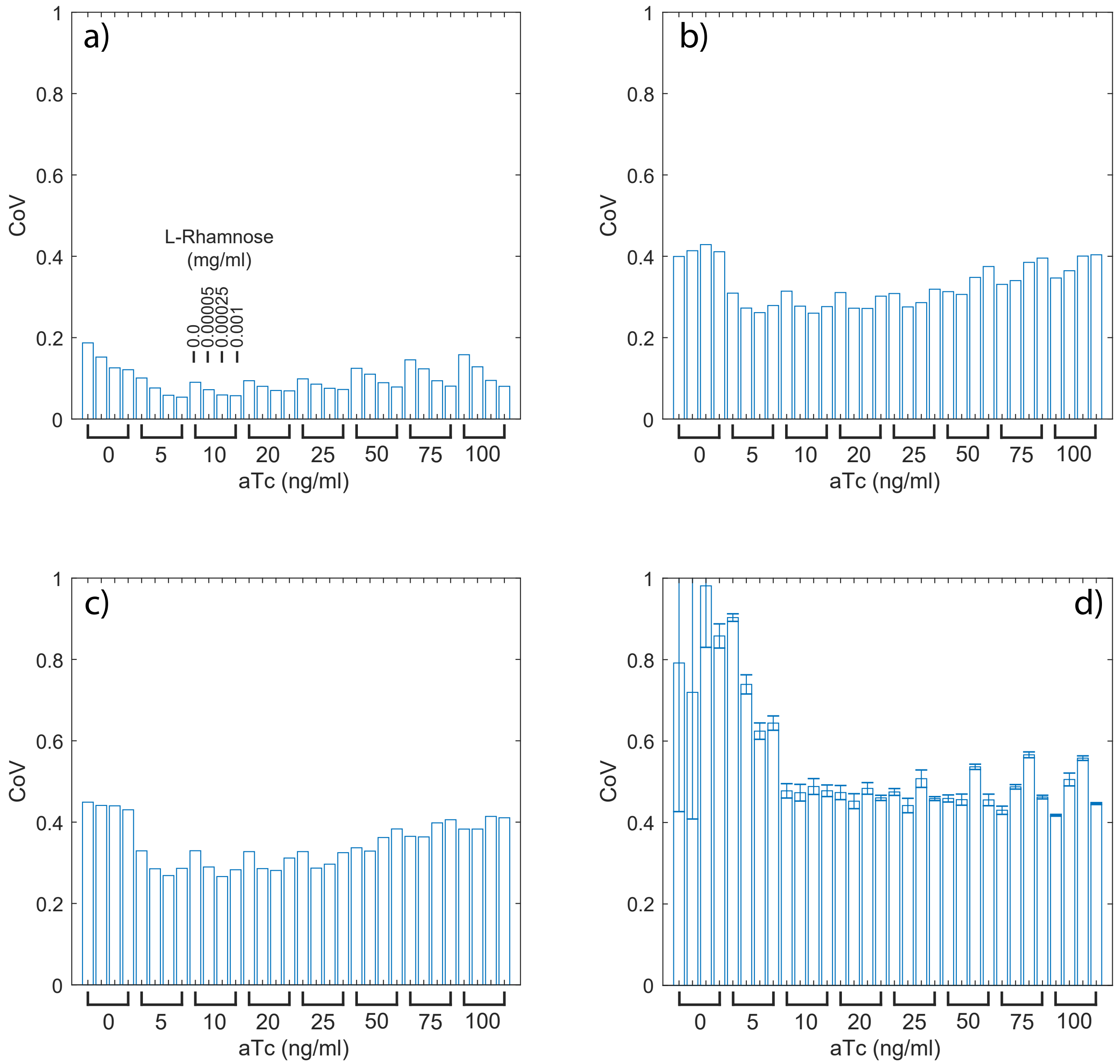

Figure S7: **sRNA-tuned Autorepressor noise simulations.** Plots of coefficient of variation (CoV) calculated as in (10) for different noise sources, and experimental data. **a)** Simulation of intrinsic noise, due to stochasticity of cellular processes. **b)** Simulation of extrinsic noise, with parameters sampled from log-normal distributions as described by (12) with $\sigma = 0.4$. **c)** Combination of stochastic and extrinsic simulations. **d)** Experimentally-measured CoV, as presented in Fig. 3 of the main text.

S1.5 Closed-loop sRNA Feedback Circuit

S1.5.1 Simple Model

To investigate the anticipated behaviour of this circuit prior to experimental implementation we again designed a simple one-equation model of our system:

$$\frac{dX}{dt} = \frac{u_3}{u_3 + 1} \times \frac{\beta}{1 + \left(\frac{u_2 X}{1+X}\right)} - X \tag{13}$$

which combines the influence of u_2 (governing the activating transcription factor's activity and hence sRNA expression) and u_3 (which determines the translation rate of the transcription factor's mRNA) into a single equation. We calculate steady-state concentrations of this model in Fig. 1b of the main manuscript, and simulate noise using the parameter β as described in Section S1.3.1. We find that together the two inputs are able to tune the system's response over a wide range, and that CoV is anticipated to remain approximately constant (or decrease) as u_2 is increased. To demonstrate the effect of feedback in this circuit we also investigate a model in which the activating transcription factor's concentration is decoupled from sRNA expression, given by:

$$\frac{dX}{dt} = \frac{u_3}{u_3+1} \times \frac{\beta}{1+u_2} - X \tag{14}$$

for which results are presented in Fig. S8. This simple simulation demonstrates qualitative differences in the steady-state behaviour of the open- and closed-loop systems as inducer concentrations vary. Namely, the output of the closed-loop system is independent of u_2 when u_3 is small. We observe similar (closed-loop system) behaviour experimentally when u_3 is small: note that in Fig. S9b the system output is largely independent of L-rhamnose when the m-toluic acid concentration is 0.01 mM.

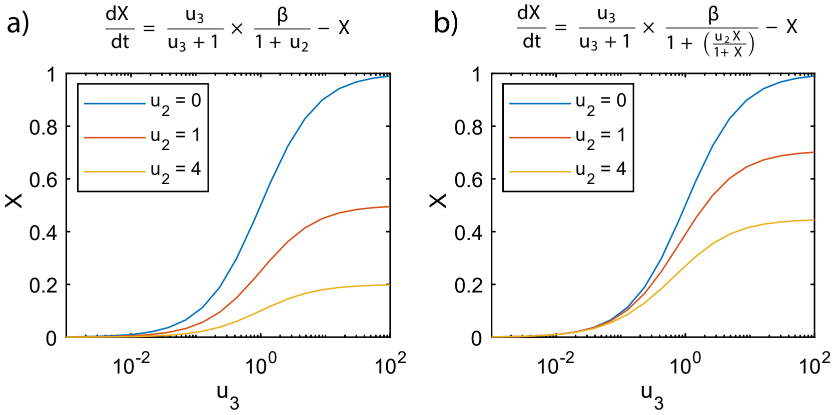

Figure S8: **Simple model of the closed-loop sRNA feedback circuit.** **a)** The open-loop system, in which the concentration of X does not impact its own rate of expression. **b)** Closed loop (feedback) system. We observe that in the closed-loop system the value of u_2 has no impact on the output when u_3 is small, whereas for the open-loop system the impact of u_2 is independent of u_3.

S1.5.2 Detailed Model

We employ the structures outlined in Section S1.1 to create a model for the closed-loop sRNA feedback circuit (Fig. S1b) that consists of four state variables: The concentration of mRNA (r) and protein (R) for the RhaS-GFP complex, the concentration of sRNA (s), and the concentration of the sRNA-mRNA complex (c). The underlying biochemical reactions can be expressed as:

$$\emptyset \xrightarrow{\gamma_X^*} \mathrm{r} \xrightarrow{\delta_r} \emptyset,$$

$$\emptyset \xrightarrow{\gamma_R^*} \mathrm{s} \xrightarrow{\delta_s} \emptyset,$$

$$\mathrm{t} + \mathrm{s} \xrightarrow{K_2} \mathrm{c} \xrightarrow{\delta_c} \emptyset,$$

$$\mathrm{r} \xrightarrow{\beta_R} \mathrm{r} + \mathrm{R},$$

$$\mathrm{R} \xrightarrow{\delta_R} \emptyset,$$

These reactions can be described using four coupled differential equations of the form:

$$
\begin{aligned}
\dot{r} &= \gamma_X^* - \delta_r r - K_2 rs \\
\dot{s} &= \gamma_R^* - \delta_s s - K_2 rs \\
\dot{c} &= K_2 rs - \delta_c c \\
\dot{R} &= \beta_R r - \delta_R R
\end{aligned}
\tag{15}
$$

S1.5.3 Parameter Fitting

We begin by fitting parameters to our circuit's behaviour in open-loop when the sRNA is not induced (no L-rhamnose is present), which allows (15) to be simplified by setting c and s to zero. The two free parameters are β_R and K_{u_3}, though we could have equivalently taken a value for β_R from the literature and fit α_x^*. The least-squares model fit for these parameters is illustrated in Fig. S9a, demonstrating that the promoter model in (4) is able to accurately capture the XylS induction system's open-loop behaviour.

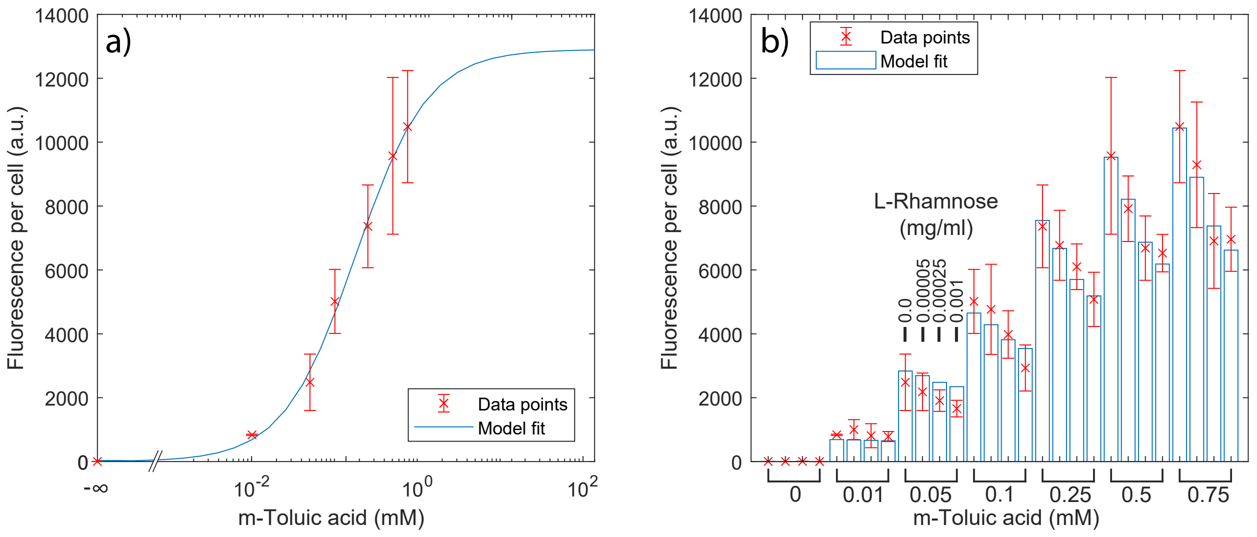

Figure S9: **Closed-loop sRNA feedback circuit model fitting. a)** Fitting of the open-loop system (L-rhamnose = 0) is used to estimate the parameters β_R and K_{u_3} in (4). We observe good agreement across the inducer range considered. **b)** Model fit for all combinations of inducer inputs. In this case we least-squares fit the values of K_2, $K_{u_2}^*$ and K_R, and set $\alpha_r^* = \alpha_r$. Resulting parameter values are summarised in Table S1.

Four parameters remain to be fit to experimental data, $K_{u_2}^*$, K_2, K_R, and α_r^*. There is a large amount of uncertainty when fitting the later three parameters: We find that values of K_R are generally large compared to the concentration of R (i.e. the model is far from saturation in rhaS concentration), and hence (2) has an approximately constant term of α_r^*/K_R. These two values can therefore vary together whilst maintaining a similar sRNA level, which is then constrained relative to r by K_2. To address this we fix α_r^* to the value of α_r found in Section S1.4, and least-squares fit $K_{u_2}^*$, K_2, and K_R to the experimental data. The results are presented in Fig. S9b, demonstrating good agreement between our model and the experimental data.

S1.5.4 Dynamic Behaviour

To assess the dynamic behaviour of the closed-loop sRNA feedback circuit we repeat a similar dynamic analysis to Section S1.4, again employing (9) to vary inducer concentrations over time. Results for these simulations are presented in Fig. S10. Similar to the tuned Autorepressor circuit we observe that it is possible to reduce the system output's sensitivity to one input, whilst maintaining a given mean output, by using the other input as a tuning dial. We observe that this circuit is much less sensitive to equivalent

fold-changes in u_2 compared to u_3. This arises due to the smaller dynamic range of induction by u_2, which in Fig. S9 is observed to typically only reduce system output by $\sim 30\%$ between zero and maximal induction.

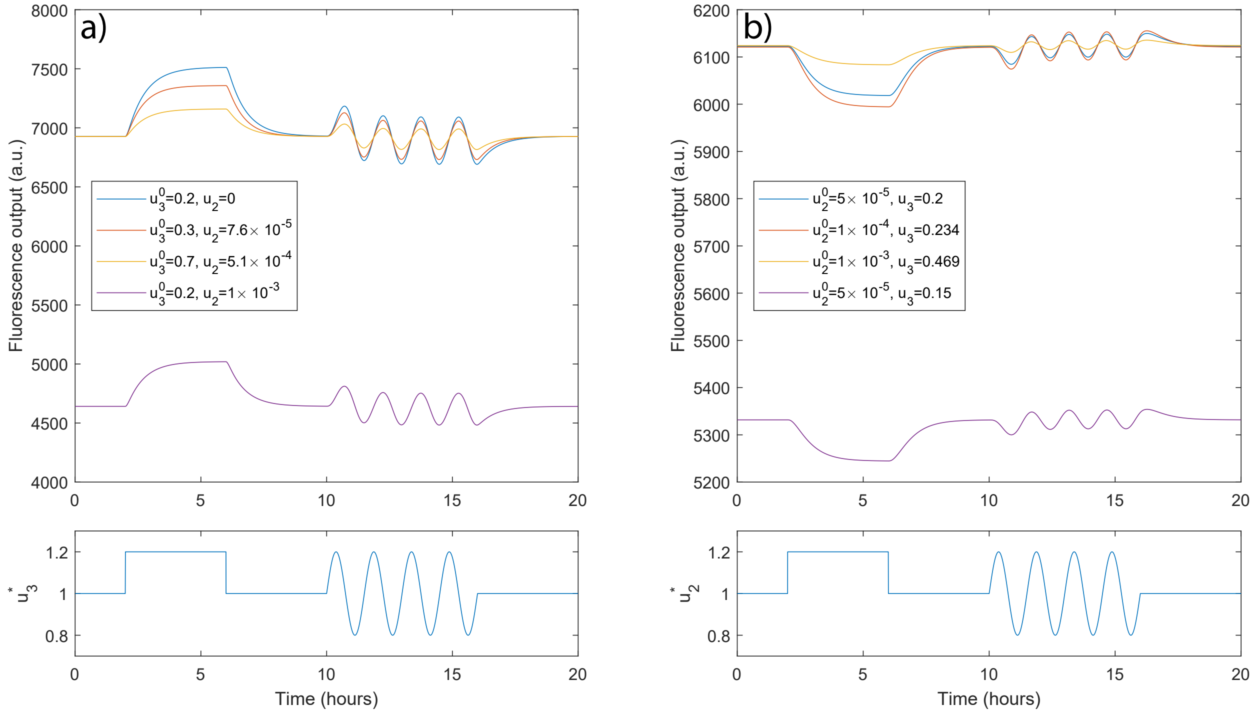

Figure S10: **Closed-loop sRNA feedback circuit dynamic simulations.** All inducer concentrations are in ng ml^{-1}. **a)** System output over time as u_3 (m-toluic acid) is varied according to (9) with the u_3^* profile illustrated. **b)** Similar to (a) but with u_2 (L-rhamnose) varying over time. We observe a slower response to changes in u_2, and smaller output fluctuations for a given fold-change in this inducer.

S1.5.5 Noise Simulation

We perform simulations of the impact of both intrinsic and extrinsic noise on our system (15) similarly to as described in Section S1.4.5. In this case we set $\sigma = 1$, as a substantially greater magnitude of variability was measured for this circuit (discussed in the main text), and we again vary the translation rate and each inducer concentration:

$$\beta_R^* = \Delta_1 \times \beta_R,\ u_{2,3}^* = \Delta_2 \times u_{1,2} \tag{16}$$

Results are presented in Fig. S11. We find again that the contribution of intrinsic noise is low compared to that of extrinsic sources, and that the trends produced by extrinsic noise simulations qualitatively replicate those measured experimentally. Our model predicts zero output (and hence zero variation) when the concentration of m-toluic acid is zero, whereas the experimental data shows a non-zero CoV in this case likely due to cellular auto-fluorescence.

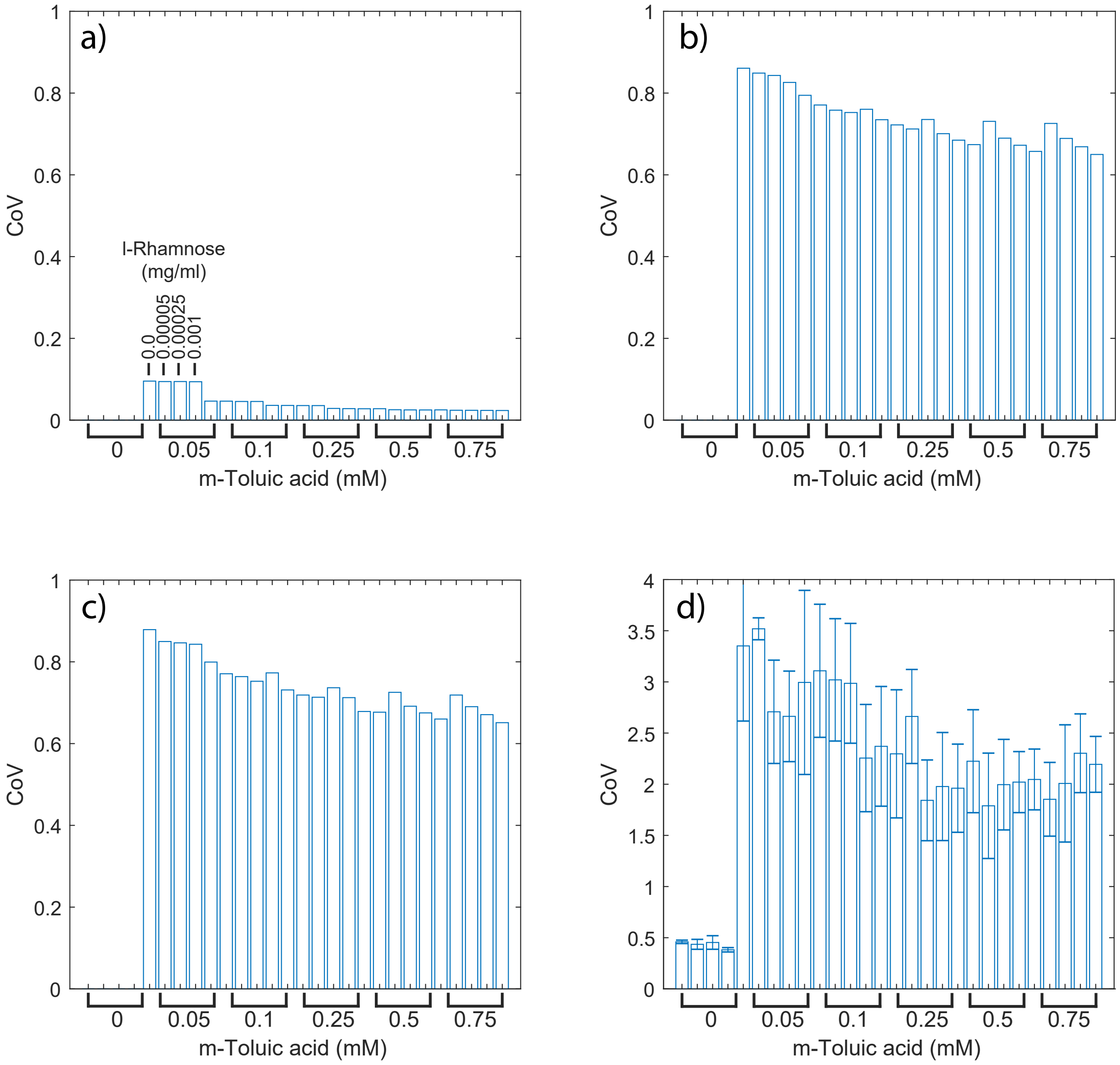

Figure S11: **Closed-loop sRNA feedback circuit noise simulations.** Plots of coefficient of variation (CoV) calculated as in (10) for different noise sources, and experimental data. **a)** Simulation of intrinsic noise, due to stochasticity of cellular processes. **b)** Simulation of extrinsic noise, with parameters sampled from log-normal distributions as described by (12) with $\sigma = 1.0$. **c)** Combination of stochastic and extrinsic simulations. **d)** Experimentally measured CoV, as presented in Fig. 4 of the main text.

S2 Additional Supplementary Materials and Methods

S2.1 Plasmid Construction

To construct the initial autorepressor plasmid, pCK200, oligonucleotides oligoCK89 and oligoCK90 were used to amplify the pCK301 backbone and a synthetic gene fragment - containing the tet promoter, a synthetic RBS with a translation initiation rate of 50,000 (designed using Salis RBS calculator [35]) and the *tetR* gene lacking its last three nucleotides (TAA). This construct was inserted in such a way to replace the first three nucleotides of *sfGFP* to ensure a TetR-sfGFP fusion protein results from translation. To construct the *rhaS*-containing version of the autorepressor plasmid, pCK210, oligonucleotides oligoCK173 and oligoCK174 were used to amplify the rhaS RBS and gene from *E. coli* strain MG1655; oligonucleotides oligoCK171 and oligoCK172 were used to amplify the pCK200 backbone and the two fragments combined (replacing P_{tet} and *tetR*).

To construct the sRNA-expressing plasmid pCK209, an intermediate plasmid pCK201 was initially constructed. A synthetic sRNA gene fragment was designed based on the pWAS plasmid [7]. The probable Shine Dalgarno sequence of the synthetic RBS of the tetR mRNA was identified (AGTAGGT) and the reverse complement of the 24 nucleotides from the start of this sequence used as the anti-tetR mRNA targeting sequence (TAATCTGGACATTCTGAACCTACT). This sequence was analysed for off target binding using IntaRNA [36] and placed immediately upstream of the the Hfq-binding scaffold of the micC sRNA from *E. coli*, followed by the T1/TE transcriptional terminator resulting in the final sRNA-expressing DNA fragment. Finally the proD promoter [37] and λ pR promoters [38] were inserted upstream of this sRNA-encoding sequence and the entire double promoter-sRNA-scaffold construct synthesised. This synthetic gene fragment was inserted into the backbone of the pSUtat-Sh-GX-EF plasmid via Gibson assembly [39], which was linearised using oligonucleotides, oligoCK87 and oligoCK88. The resulting plasmid pCK201 was modified to remove the unwanted λ pR promoter by amplification using oligonucleotides, oligoCK127 and oligoCK124 and blunt-end ligation of the resulting PCR fragment resulting in pCK209. The alternative anti-tetR mRNA sRNA plasmid, pCK220, was constructed similarly, but the 24 antisense nucleotides chosen to bind to the start codon of the tetR mRNA. This targeting sequence was analysed for off target binding, combined with same promoters and scaffold sequences, synthesised and inserted into the same location of pSUtat-Sh-GX-EF backbone resulting in the intermediate plasmid pCK202. The unwanted λ pR promoter was removed from the resulting plasmid pCK202, through amplification using oligonucleotides, oligoCK126 and oligoCK600 and blunt-end ligation of the PCR fragment, resulting in plasmid pCK220. The control plasmid, pAH23 was constructed by removing the section containing the promoters, sRNA and terminator from pCK201 through amplification of the backbone into two fragments using oligonucleotides: oligoAH007 and oligoAH008; and oligoAH009 and oligoAH010, which were subsequently combined resulting in the final plasmid. pAH17 was constructed using oligonucleotides oligoAH001 and oligoAH013 to amplify the *rhaBAD* promoter from pAH16 and oligoAH12 and oligoAH040 to replace the two promoters and amplify the sRNA-encoding sequences and backbone of pCK201, which were then assembled to form the finished plasmid.

The combined L-rhamnose inducible sRNA-tuned autorepressor plasmid pCK221 was constructed as follows. Oligonucleotides oligoCK449 and oligoCK104 were used to amplify the backbone of pCK210 and oligonucleotides oligoCK451 and oligoCK450 used to amplify the *rhaBAD* promoter-sRNA-scaffold sequence from pAH17. The resulting PCR products were combined, resulting in the intermediate plasmid pCK214. The *lacI* gene was removed by amplification of pCK214 with oligonucleotides oligoCK324 and oligoCK100 and the resulting PCR product recircularised using blunt-end ligation yielding intermediate plasmid pCK215. pCK215 was further modified to remove five unwanted nucleotides located immediately downstream of the transcriptional start site of the *rhaBAD* promoter which could affect the binding of the sRNA sequence to the tetR mRNA RBS. Oligonucleotides oligoCK124 and oligoCK490 were used to amplify pCK215 and blunt-end ligation was used to recircularise the PCR product, resulting in the final plasmid pCK221. The alternative *m*-toluic acid inducible sRNA-tuned autorepressor plasmid pCK226 was constructed as follows. A fragment containing the *xylS* gene and the Pm promoter was amplified from the expression vector pSEVA238 (a gift from Victor de Lorenzos lab)[40] using 5-phosphorylated oligonucleotides oligoCK705 and oligoCK708; pCK221 was amplified using oligonucleotides oligoCK124 and oligoCK104 and the two fragments ligated

using blunt-end ligation resulting in the finished plasmid pCK226.

To construct the *rhaS*-expressing plasmid pAH12, the proD promoter from pCK201 was amplified using oligonucleotides, oligoAH003 and oligoAH005, the *rhaS* RBS and gene was amplified from pCK210 using oligonucleotides, oligoAH004 and oligoAH006, and both were inserted into the same backbone PCR product as used for pCK200 construction (from pCK301). To construct the anti-rhaS mRNA sRNA plasmids pAH15 and pAH16, oligonucleotides oligoAH001 and oligoCK88 were used to amplify the backbone of pCK209 and a synthetic gene fragment encoding an sRNA targeting a 24 base pair region of the rhaS mRNA beginning with the start codon in the case of pAH15 and the Shine Dalgarno sequence in the case of pAH16 of the rhaS mRNA, was designed, synthesised and inserted into this backbone fragment. These were analysed for off target binding as before, and contain the same Hfq-binding and terminator scaffold sequences as in pCK209, pCK220 etc. The *rhaBAD* promoter and SD-targeting sRNA sequences were inserted into the plasmid encoding RhaS, pAH12, as follows. Oligonucleotides oligoCK449 and oligoCK104 were used to amplify the backbone of pAH12 and oligoCK451 and oligoCK450 used to amplify the promoter-sRNA-scaffold sequence from pAH16, and the resulting PCR products combined to yield intermediate plasmid pCK218. This plasmid was modified further to remove five unwanted nucleotides located immediately downstream of the transcriptional start site of the *rhaBAD* promoter which could affect the binding of the sRNA sequence to the rhaS mRNA RBS. Oligonucleotides oligoCK490 and oligoCK497 were used amplify pCK218 and blunt-end ligation used to recircularise the PCR product, resulting in plasmid pCK222. Finally the constitutive proD promoter upstream of *rhaS* was replaced with the *Pm* promoter, allowing induction of the anti-tetR SD sRNA in the presence of the transcriptional activator XylS and *m*-toluic acid. A fragment containing the *xylS* gene and the *Pm* promoter was amplified from the expression vector pSEVA238 (a gift from Victor de Lorenzos lab) using oligonucleotides oligoCK620 and oligoCK621 and pCK222 was amplified using oligonucleotides oligoCK623 and oligoCK622 and the two fragments combined using Gibson assembly resulting in the finished plasmid pCK227.

S2.2 Assays

For platereader experiments, overnight cultures were subcultured into 150 L of fresh media in 96-well microplates, to an optical density (OD; A600 nm) of 0.05 and the plate incubated at 37 C with rapid orbital shaking (platereader preset speed: high) in a BioTek Synergy HT platereader (Biotek Instruments). Optical density (measured at 600 nm) was measured every 15 minutes. Error bars shown represent the standard deviation of three independent biological replicates.

S2.3 Supplementary Results

The use of an RNA-based controller instead of a protein-based controller in negative feedback circuits is likely to reduce burden on the cell, as the costly high-level production of a protein that is turned over is avoided. Although not the focus of this work, we wanted to briefly test this hypothesis by comparing the growth curves of cells containing each of our sRNA-based feedback circuits when the sRNA was expressed or not. Cells containing either: pCK210 and one of pAH23, pCK209, pCK220; pCK221; or pCK222, were grown in rich defined medium in a microplate reader and the optical density (measured at 600 nm) of cells monitored over time (Fig. S15). No difference was observed between the growth curves of cells containing the autorepressor plasmid in combination with either the constitutive SD- or Start-targeting sRNA plasmids (pCK209 or pCK220) or an empty control plasmid (pAH23) (Fig. S15a). The promoter used to express the sRNA is a very strong promoter and so it is encouraging that the expression of the sRNA did not result in growth defects. Cells containing the combined sRNA-tuned autorepressor plasmid, pCK221 did not show any statistically-significant difference in growth when aTc or L-rhamnose was supplied (Fig. S15b). Cells containing the closed-loop sRNA feedback circuit, pCK222 did not show any statistically-significant difference in growth when sRNA expression was induced with L-rhamnose (Fig. S15c). All three of these results support the hypothesis that sRNA expression does not impart a large burden on the cell, as the production of a short (approximately 200 bp nucleic-acid oligomer) requires less cellular resources than a large amino-acid polypeptide such as a transcription factor.

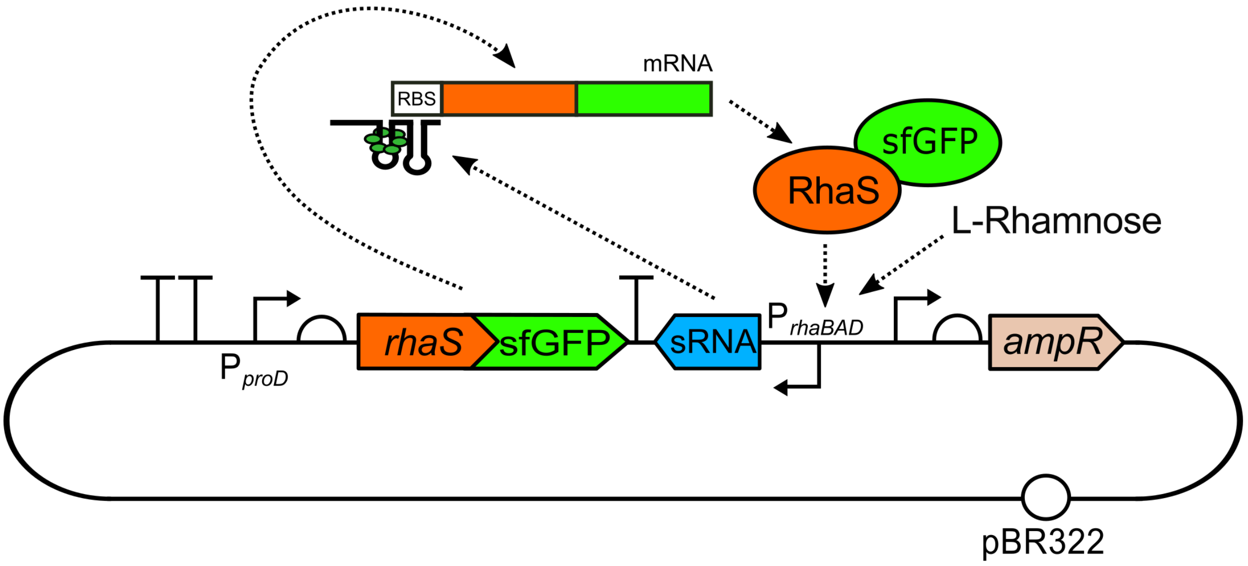

Figure S12: Schematic of plasmid pCK222 encoding the closed-loop sRNA negative feedback circuit with constitutive expression of *rhaS-sfGFP*.

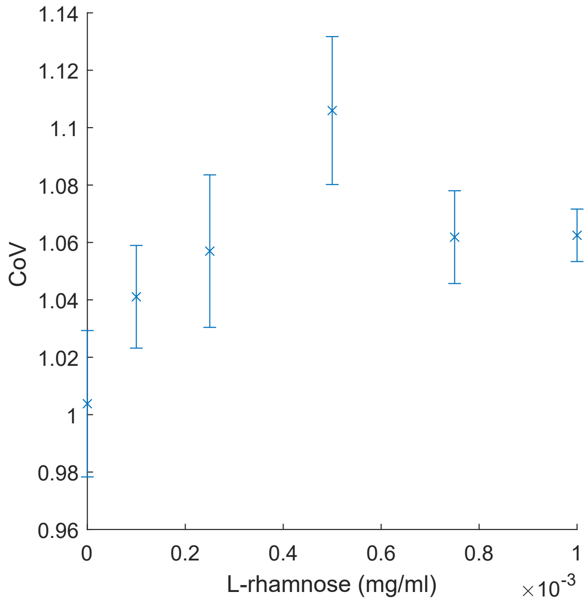

Figure S13: Experimentally-obtained coefficients of variation around the RhaS-sfGFP mean with pCK222, when output is tuned using L-rhamnose alone. Error bars shown represent the standard deviation of three independent biological replicates.

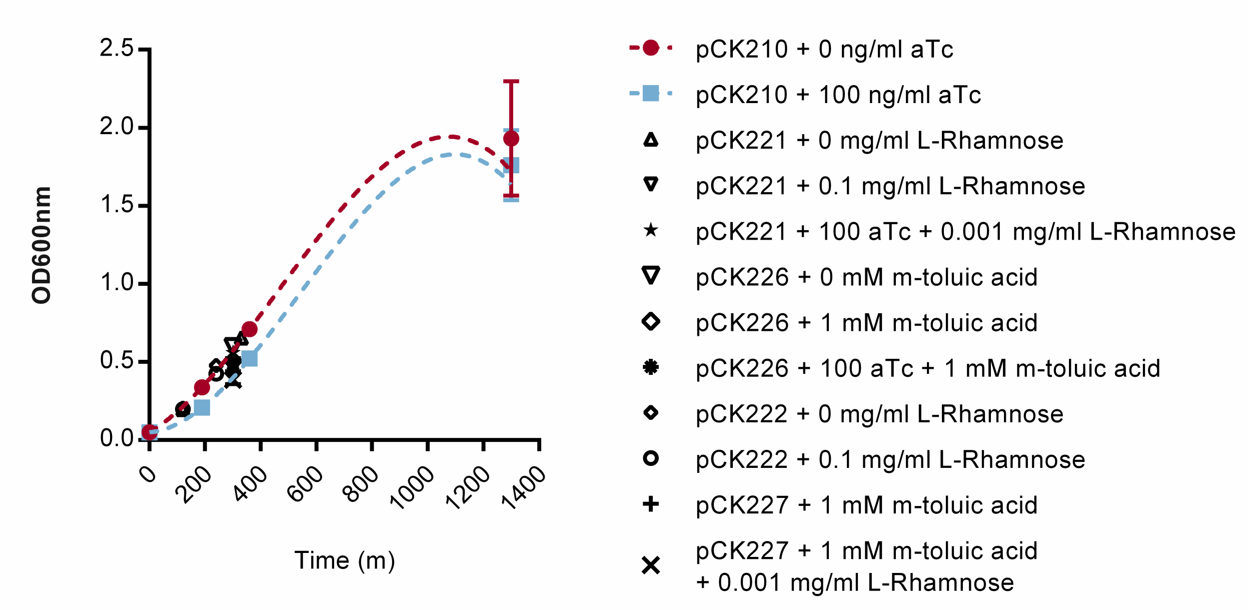

Figure S14: A comparison of cell turbidity at time of flow-cytometry sampling of all circuits. The optical density (measured at 600 nm) of JW3876 cells containing the autorepressor plasmid pCK210 and grown in rich defined medium supplemented with glycerol was measured over time and a non-linear curve (polynomial) fitted to these time points. These growth curves were compared to the optical densities of cells containing each of the sRNA-feedback circuit plasmids: pCK221, pCK226, pCK222, pCK227 grown in identical conditions with and without inducers, at the time of flow cytometry measurement. All samples were sampled at 4 or 5 h (stated in text), which consistently coincides with mid-late exponential phase of growth. Error bars shown represent the standard deviation of three independent biological replicates.

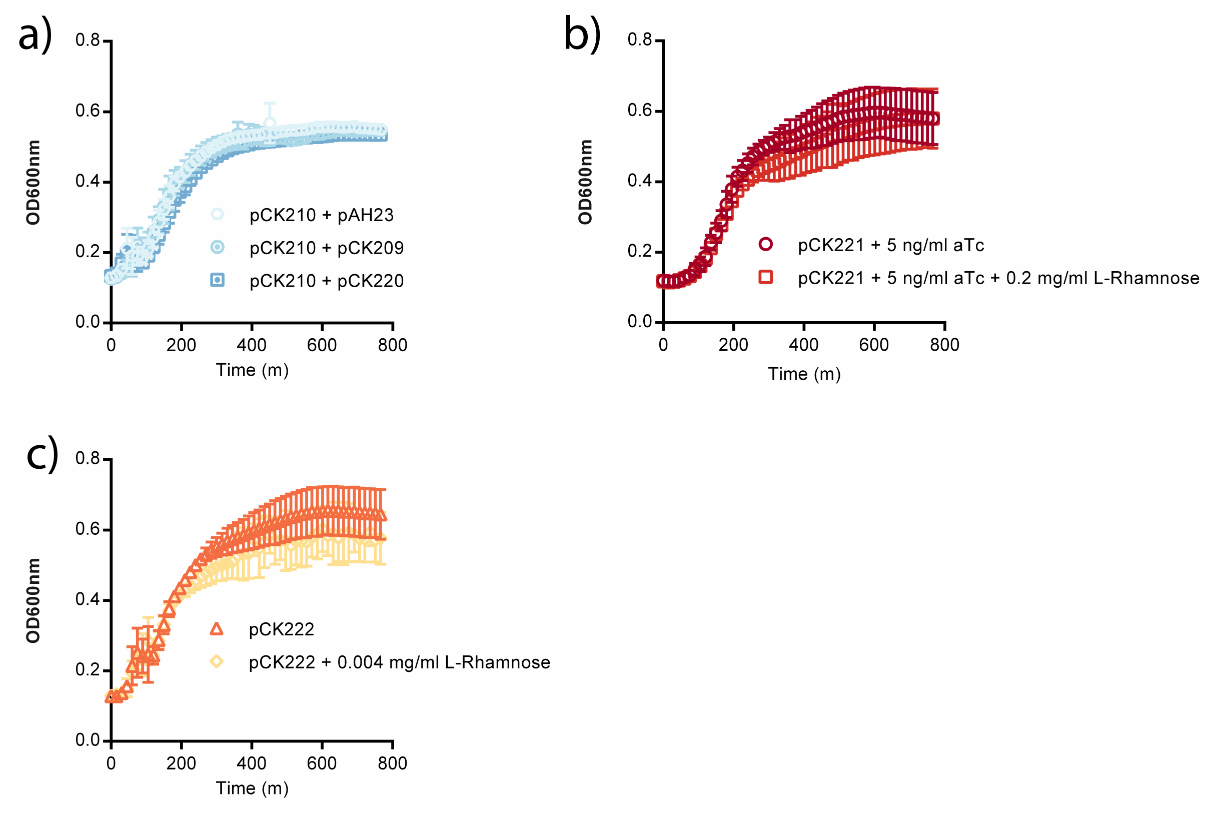

Figure S15: **Testing the effect of sRNA-expression and sRNA-based feedback circuits on cell growth.** **a)** JW3876 cells containing pCK210 and one of pAH23 (no sRNA), pCK209 (SD) or pCK220 (Start) were cultured at 37 °C in EZ rich defined medium supplemented with glycerol in a microplate reader with vigorous shaking, and optical density and fluorescence monitored over time. **b)** JW3876 cells containing pCK221 were cultured at 37 °C in EZ rich defined medium supplemented with glycerol, 5 ng/ml aTc and 0 or 0.2 mg/ml L-rhamnose (for full sRNA expression) in a microplate reader with vigorous shaking, and optical density and fluorescence monitored over time. **c)** JW3876 cells containing pCK222 were cultured at 37 °C in EZ rich defined medium supplemented with glycerol and 0 or 0.4 mg/ml L-rhamnose (ensuring feedback-loop equilibrium is reached) in a microplate reader with vigorous shaking, and optical density and fluorescence monitored over time. No differences were observed between cells expressing sRNAs and those not expressing sRNAs. Error bars shown represent the standard deviation of three independent biological replicates.

Table S2

IntraRNA analysis of each sRNA construct for target and off-target mRNA binding.

anti-tetR SD-targeting sRNA

**Putative Target**	**ΔG sRNA-mRNA binding**	**Annotation**
tetR	-37.35320	N/A
ycgB	-19.393	SpoVR family stationary phase protein
ysgA	-17.3944	putative carboxymethylenebutenolidase
yaiX	-16.7026	pseudogene interrupted by IS2A hexapeptide transferase superfamily putative transferase
ddpD	-16.5345	DD-dipeptide ABC transporter ATPase
yfgD	-16.4976	putative oxidoreductase
priB	-16.1283	primosomal protein N
yneF	-16.1113	putative membrane-bound diguanylate cyclase
murI	-15.8632	glutamate racemase
asnC	-15.6967	transcriptional activator of asnA autorepressor
yijF	-15.1121	DUF1287 family protein
zur	-14.8781	transcriptional repressor Zn(II)-binding

anti-tetR Start-targeting sRNA

**Putative Target**	**ΔG sRNA-mRNA binding**	**Annotation**
tetR	-31.09130	N/A
ycaM	-14.5851	putative transporter
yfeW	-12.5948	penicillin binding protein PBP4B weak DD-carboxypeptidase activity
waaA	-12.4821	3-deoxy-D-manno-octulosonic-acid transferase (KDO transferase)
tgt	-12.0723	tRNA-guanine transglycosylase
yehH	-12.002	DUF4132 family pseudogene defective yehI paralog molybdate metabolism regulator first fragment molybdate metabolism regulator second fragment 2 yehI' molR mutations have phenotypes and the N-terminal molR fragment of yehI' may be expressed and may have retained or evolved MolR function
ydgA	-11.8967	DUF945 family protein

asnC	-11.6628	transcriptional activator of asnA autorepressor
trmA	-11.6267	tRNA m(5)U54 methyltransferase SAM-dependent tmRNA m(5)U341 methyltransferase
holC	-11.4904	DNA polymerase III chi subunit
ridA	-11.3994	enamine/imine deaminase reaction intermediate detoxification

anti-rhaS SD-targeting sRNA

Putative Target	ΔG sRNA-mRNA binding	Annotation
rhaS	-36.0065	N/A
yaeQ	-18.7936	PDDEXK superfamily protein
ycfL	-18.3231	uncharacterized protein
ydaW	-18.2965	Rac prophage pseudogene DNA-binding protein familyPhage or Prophage Related
ygeR	-17.5273	LysM domain-containing M23 family putative peptidase septation lipoprotein
yebB	-17.2584	DUF830 family protein
ybbJ	-16.4493	inner membrane protein stimulator of the QmcA suppressor of ftsH-htpX
ypfN	-16.4485	putative membrane protein UPF0370 family
yceG	-16.2901	septation protein ampicillin sensitivity
wzxE	-16.1767	O-antigen translocase
dxs	-16.1273	1-deoxyxylulose-5-phosphate synthase thiamine triphosphate-binding FAD-requiring
gspM	-16.1088	general secretory pathway component cryptic
yqiG	-16.1087	pseudogene fimbrial export usher familyputative membrane Not classified putative membrane protein
nfrB	-15.9668	bacteriophage N4 receptor inner membrane subunit
yhhT	-15.9564	UPF0118 family putative transporter
insJ	-15.9123	IS150 transposase A
dnaG	-15.6289	DNA primase
yfeH	-15.5533	putative inorganic ion transporter
gfcA	-15.494	O-antigen capsule production threonine-rich inner membrane protein
fkpA	-15.4147	FKBP-type peptidyl-prolyl cis-trans isomerase (rotamase)

hisJ	-15.354	histidine ABC transporter periplasmic binding protein
wecG	-15.3467	UDP-N-acetyl-D-mannosaminuronic acid transferase
aroP	-15.3205	aromatic amino acid transporter
dmlA	-15.319	D-malate oxidase NAD-dependent putative tartrate dehydrogenase
ygeN	-15.3114	pseudogene orgB family part of T3SS PAI ETT2 remnant
zapE	-15.1199	divisome ATPase
tfaD	-15.1161	pseudogene DLP12 prophage tail fiber assembly protein familyPhage or Prophage Related

anti-rhaS Start-targeting sRNA

**Putative Target**	**ΔG sRNA-mRNA binding**	**Annotation**
rhaS	-33.5296	N/A
yahL	-15.6609	uncharacterized protein
dppF	-13.9628	dipeptide/heme ABC transporter ATPas
rtn	-13.786	resistance protein for phages lambda and N4 putative membrane-anchored cyclic-di-GMP phosphodiesterase
rcsC	-13.6385	hybrid sensory kinase in two-component regulatory system with RcsB and YojN
ymjC	-13.413	pseudogene
pdxY	-13.2001	pyridoxamine kinase
yacL	-12.3295	UPF0231 family protein
dnaC	-12.2886	DNA biosynthesis protein
rpmA	-12.2046	50S ribosomal subunit protein L27
yjhI	-12.1904	putative DNA-binding transcriptional regulator

Table S3

Plasmids and oligonucleotides used in this study.

Name	Details
pCK301	As pCK300 with *E. coli rhaBAD* promoter inserted upstream of *sfGFP* gene (78).
pCK302	As pCK301 with *E. coli rhaS* inserted downstream of *ampR* (78).
pSUtat-Sh-GX-EF	Medium copy (p15A origin) plasmid with *CmR* containing *hydE*, *hydF* and *hydG* from *S. oneidensis* (79).
pCK200	As pCK301 with the tetracycline-response *tet* operon promoter (*tet* promoter), synthetic RBS and the Tn10 *tetR* gene inserted upstream of the *sfGFP* gene, resulting in a translational fusion of TetR and sfGFP.
pCK201	As pSUtat-Sh-GX-EF backbone but with two constitutive promoters proD and λ pR and DNA encoding a chimeric sRNA targeting the Shine Dalgarno of the tetR mRNA inserted in place of *hydE*,*F*,*G*.
pCK202	As pCK201 but encoding a chimeric sRNA that binds to the start codon of the tetR mRNA instead of the Shine Dalgarno sequence.
pCK209	As pCK201 but with the λ pR promoter removed.
pAH12	As pCK301 but with the proD promoter and the *E. coli rhaS* gene inserted upstream of the *sfGFP* gene, resulting in a translational fusion of RhaS and sfGFP.
pAH15	As pCK201 but with the *E. coli rhaBAD* promoter and DNA encoding a chimeric sRNA targeting the start codon of rhaS mRNA in place of anti-tetR sequence.
pAH16	As pCK201 but with the *E. coli rhaBAD* promoter and DNA encoding a chimeric sRNA targeting the Shine Dalgarno sequence of rhaS mRNA in place of anti-tetR sequence.
pAH17	As pCK201 but with the *E. coli rhaBAD* promoter in place of the two constitutive promoters.

pCK210	As pCK200 but with the *E. coli rhaS* RBS and gene inserted downstream of the *ampR* gene.
pCK214	As pCK210 but with the *rhaBAD* promoter and sRNA-encoding sequence from pAH17 inserted between the *rrnB1 B2* T1 terminator and *ampR*.
pCK215	As pCK214 but with the *lacI* gene deleted.
pCK221	As pCK215 but with the unwanted extra 5 bases downstream of the TSS+1 of the *rhaBAD* promoter removed.
pCK226	As pCK221 but with *xylS* and *Pm* promoter replacing the *rhaBAD* promoter allowing *m*-toluic acid induction of sRNA
pCK218	As pAH12 but with the *rhaBAD* promoter and sRNA-encoding sequence from pAH16 inserted between the *rrnB1 B2* T1 terminator and *ampR*.
pCK222	As pCK218 but with the unwanted extra 5 bases downstream of the TSS+1 of the *rhaBAD* promoter removed.
pCK227	As pCK218 but with *xylS* and *Pm* promoter replacing the proD promoter allowing *m*-toluic acid induction of *rhaS-sfGFP*.
pAH23	As pCK201 but with promoters, sRNA and terminator removed. Used as control plasmid.
oligoCK87	acgacgtggtgttagctgtgCAATTCACTGGCCGTCGTTTTACAACGTCG
oligoCK88	tgggcctttctgcgtttataATTGCGTTGCGCTCACTGCCC
oligoCK89	cgtaaaggcgaagagctgttcactgg
oligoCK90	aagggcgacacaaaatttattctaaatg
oligoCK100	ccaaaatcccttaacgtgagttacg
oligoCK104	gctcgcgataatgttcagaattgg
oligoCK120	taacaccgtgcgtgttgactattttacctctggcggtgataatgg
oligoCK121	gtcaacacgcacggtgttaCAATTCACTGGCCGTCGTTTTACAACG

oligoCK124	taatctggacatTCTGAACCTACTtttctgttgggcc
oligoCK127	gcaatggcccaacagaaaatgtccagattagataaaagtaaaaaagttaaacaaaattatttgtagaggg aaaccg
oligoCK173	ggtgcctcactgattaagcattggtaagcggcgcgccaTATTTCGCCGTGTTGACGAC
oligoCK174	cgctatcatgccataccgcgaaaggttttgcgccattcgattattgcagaaagccatcc
oligoCK171	tcgaatggcgcaaaacctttcgcggtatggcatgatagcg
oligoCK172	tggcgcgccgcttaccaatgcttaatcagtgaggcacc
oligoCK324	attcaccaccctgaattgactctcttcc
oligoCK449	ccatttatacctgaatatggctcataacaccc
oligoCK450	gggtgttatgagccatattcaggtataaatggtataaacgcagaaaggcccacccgaagg
oligoCK451	ccaattctgaacattatcgcgagcccacaattcagcaaattgtgaacatcatcacg
oligoCK490	tacgaccagtctaaaaagcgcc
oligoCK497	ACTGGCCTCCTGATGTCGTCAACAtttctgttgggcc
oligoCK600	aaagttaaacaaaattatttgtagagggaaaccg
oligoCK620	tgcatttagaataaattttgtgtcgccctttcaagccacttcctttttgcattgacgcag
oligoCK621	GTCGTCAACACGGCGAAATAttgcataaagcctaaggggtaggccttactagagatagc
oligoCK622	aagggcgacacaaaatttattctaaatgcataataaatactgataaca
oligoCK623	TATTTCGCCGTGTTGACGACATCAGGaggccagtatgaccgtattacatagtgtgg
oligoCK705	tcaagccacttcctttttgcattgacg
oligoCK708	ttgcataaagcctaaggggtaggc
oligoAH001	CAATTCACTGGCCGTCGTTTTACAACGTCG
oligoAH003	catttagaataaattttgtgtcgcccttcacagctaacaccacg

oligoAH004	ccctctacaaataattttgtttaactttTATTTCGCCGTGTTGACGACATCAGG
oligoAH005	CCTGATGTCGTCAACACGGCGAAATAaaagttaaacaaaattatttgtagaggg
oligoAH006	gaatagggacgacaccagtgaacagctcttcgcctttacgTTGCAGAAAGCCATCCC
oligoAH007	ATTGCGTTGCGCTCACTGCCC
oligoAH008	GGGCAAATATTATACGCAAGGCGACAAGGTGCTGATGCCGCTGGC
oligoAH009	GCCAGCGGCATCAGCACCTTGTCGCCTTGCGTATAATATTTGCCC
oligoAH010	GGGCAGTGAGCGCAACGCAATCAATTCACTGGCCGTCGTTTTACAACGTCG
oligoAH012	AGTAGGTTCAGAatgtccagattattcattacgaccagtctaaaaagcgcc
oligoAH013	ggcgctttttagactggtcgtaatgaataatctggacatTCTGAACCTACT
oligoAH040	CGACGTTGTAAAACGACGGCCAGTGAATTGccacaattcagcaaattgtg

Supplementary References

[1] T. Ellis, X. Wang, and J. J. Collins, "Diversity-based, model-guided construction of synthetic gene networks with predicted functions," *Nature Biotechnology*, vol. 27, no. 5, pp. 465–471, 2009.

[2] C. H. Wu, H. C. Lee, and B. S. Chen, "Robust synthetic gene network design via library-based search method," *Bioinformatics*, vol. 27, no. 19, pp. 2700–2706, 2011.

[3] D. Braun, S. Basu, and R. Weiss, "Parameter estimation for two synthetic gene networks: A case study," *Proceedings of IEEE International Conference on Acoustics, Speech, and Signal Processing, 2005*, vol. 5, pp. 769–772, 2005.

[4] M. B. Elowitz and S. Leibler, "A synthetic oscillatory network of transcriptional regulators," *Nature*, vol. 403, pp. 335–338, jan 2000.

[5] B. P. Ingalls, *Mathematical Modelling in Systems Biology : An Introduction.* MIT Press, 2014.

[6] A. W. K. Harris, H. Steel, C. L. Kelly, and A. Papachristodoulou, "The Autorepressor: a Case Study of the Importance of Model Selection," *Proceedings of the 56th IEEE Conference on Decision and Control.*, pp. 1622–1627, 2017.

[7] D. Na, S. M. Yoo, H. Chung, H. Park, J. H. Park, and S. Y. Lee, "Metabolic engineering of Escherichia coli using synthetic small regulatory RNAs.," *Nature biotechnology*, vol. 31, no. 2, pp. 170–4, 2013.

[8] D. N. Adamson and H. N. Lim, "Essential requirements for robust signaling in Hfq dependent small RNA networks," *PLoS Computational Biology*, vol. 7, no. 8, 2011.

[9] T. Soper, P. Mandin, N. Majdalani, S. Gottesman, and S. a. Woodson, "Positive regulation by small RNAs and the role of Hfq," *Pnas*, vol. 107, no. 21, pp. 2–7, 2010.

[10] J. F. Hopkins, S. Panja, and S. A. Woodson, "Rapid binding and release of Hfq from ternary complexes during RNA annealing," *Nucleic Acids Research*, vol. 39, no. 12, pp. 5193–5202, 2011.

[11] E. G. H. Wagner, "Cycling of RNAs on Hfq," *RNA Biology*, vol. 10, no. 4, pp. 619–626, 2013.

[12] Y. Dublanche, K. Michalodimitrakis, N. Kummerer, M. Foglierini, and L. Serrano, "Noise in transcription negative feedback loops: simulation and experimental analysis," *Mol Syst Biol*, vol. 2, p. 41, 2006.

[13] D. Kennell and H. Riezman, "Transcription and translation initiation frequencies of the Escherichia coli lac operon," *Journal of Molecular Biology*, vol. 114, no. 1, pp. 1–21, 1977.

[14] S. T. Liang, M. Bipatnath, Y. C. Xu, S. L. Chen, P. Dennis, M. Ehrenberg, and H. Bremer, "Activities of constitutive promoters in Escherichia coli," *Journal of Molecular Biology*, vol. 292, no. 1, pp. 19–37, 1999.

[15] H. Chen, K. Shiroguchi, H. Ge, and X. S. Xie, "Genome-wide study of mRNA degradation and transcript elongation in Escherichia coli," *Molecular Systems Biology*, vol. 11, no. 1, pp. 781–781, 2015.

[16] C. A. McCullen, J. N. Benhammou, N. Majdalani, and S. Gottesman, "Mechanism of positive regulation by DsrA and RprA small noncoding RNAs: Pairing increases translation and protects rpoS mRNA from degradation," *Journal of Bacteriology*, vol. 192, no. 21, pp. 5559–5571, 2010.

[17] R. Guantes, B. Cayrol, F. Busi, and V. Arluison, "Positive regulatory dynamics by a small noncoding RNA: speeding up responses under temperature stress," *Molecular BioSystems*, vol. 8, no. 6, p. 1707, 2012.

[18] M. Elowitz, A. Levine, E. Siggia, and P. Swain, "Stochastic gene expression in a single cell," *Science*, vol. 297, no. 5584, pp. 1183–6, 2002.

[19] P. S. Swain, M. B. Elowitz, and E. D. Siggia, "Intrinsic and extrinsic contributions to stochasticity in gene expression.," *Proceedings of the National Academy of Sciences of the United States of America*, vol. 99, no. 20, pp. 12795–800, 2002.

[20] D. A. Oyarzún, J. B. Lugagne, and G. B. V. Stan, "Noise propagation in synthetic gene circuits for metabolic control," *ACS Synthetic Biology*, vol. 4, no. 2, pp. 116–125, 2015.

[21] J. L. Snoep, L. P. Yomano, H. V. Westerhoff, and L. O. Ingram, "Protein burden in Zymomonas mobilis: Negative flux and growth control due to overproduction of glycolytic enzymes," *Microbiology*, vol. 141, no. 9, pp. 2329–2337, 1995.

[22] Y. Qian and D. Del Vecchio, "Mitigation of ribosome competition through distributed sRNA feedback," *Proc. of IEEE Conference on Decision and Control*, pp. 1–30, 2016.

[23] J.-W. Veening, W. K. Smits, and O. P. Kuipers, "Bistability, Epigenetics, and Bet-Hedging in Bacteria," *Annual Review of Microbiology*, vol. 62, no. 1, pp. 193–210, 2008.

[24] L. Robert, G. Paul, Y. Chen, F. Taddei, D. Baigl, and A. B. Lindner, "Pre-dispositions and epigenetic inheritance in the Escherichia coli lactose operon bistable switch," *Molecular Systems Biology*, vol. 6, no. 357, 2010.

[25] B. Snijder and L. Pelkmans, "Origins of regulated cell-to-cell variability," *Nature Reviews Molecular Cell Biology*, vol. 12, no. 2, pp. 119–125, 2011.

[26] M. Soltani, C. A. Vargas-Garcia, D. Antunes, and A. Singh, "Intercellular Variability in Protein Levels from Stochastic Expression and Noisy Cell Cycle Processes," *PLoS Computational Biology*, vol. 12, no. 8, pp. 1–23, 2016.

[27] A. Sanchez and I. Golding, "Genetic Determinants and Cellular Constraints in Noisy Gene Expression," *Science*, no. December, pp. 1188–1193, 2013.

[28] A. Hilfinger and J. Paulsson, "Separating intrinsic from extrinsic fluctuations in dynamic biological systems," *Proceedings of the National Academy of Sciences*, vol. 108, no. 29, pp. 12167–12172, 2011.

[29] A. Singh and M. Soltani, "Quantifying intrinsic and extrinsic variability in stochastic gene expression models," *PLoS ONE*, vol. 8, no. 12, 2013.

[30] N. Rosenfeld, J. Young, U. Alon, P. Swain, and M. Elowitz, "Gene regulation at the single-cell level," *Science*, vol. 307, no. 5717, pp. 1962–1965, 2005.

[31] H. Steel and A. Papachristodoulou, "Probing InterCell Variability using Bulk Measurements," *ACS Synthetic Biology*, p. acssynbio.8b00014, 2018.

[32] M. Kaern, T. C. Elston, W. J. Blake, and J. J. Collins, "Stochasticity in gene expression: From theories to phenotypes," *Nature Reviews Genetics*, vol. 6, no. 6, pp. 451–464, 2005.

[33] J. Beal, "Biochemical complexity drives log-normal variation in genetic expression," *Engineering Biology*, vol. 1, no. 1, pp. 55–60, 2017.

[34] M. Bengtsson, A. Ståhlberg, P. Rorsman, and M. Kubista, "Gene expression profiling in single cells from the pancreatic islets of Langerhans reveals lognormal distribution of mRNA levels," *Genome Research*, vol. 15, pp. 1388–1392, 2005.

[35] H. M. Salis, E. A. Mirsky, and C. A. Voigt, "Automated design of synthetic ribosome binding sites to control protein expression," *Nature Biotechnology*, vol. 27, no. 10, pp. 946–950, 2009.

[36] A. Busch, A. S. Richter, and R. Backofen, "IntaRNA: Efficient prediction of bacterial sRNA targets incorporating target site accessibility and seed regions," *Bioinformatics*, vol. 24, no. 24, pp. 2849–2856, 2008.

[37] J. H. Davis, A. J. Rubin, and R. T. Sauer, "Design, construction and characterization of a set of insulated bacterial promoters," *Nucleic Acids Research*, vol. 39, no. 3, pp. 1131–1141, 2011.

[38] S. M. Yoo, D. Na, and S. Y. Lee, "Design and use of synthetic regulatory small RNAs to control gene expression in Escherichia coli," *Nature Protocols*, vol. 8, no. 9, pp. 1694–1707, 2013.

[39] D. G. Gibson, L. Young, R. Y. Chuang, J. C. Venter, C. A. Hutchison, and H. O. Smith, "Enzymatic assembly of DNA molecules up to several hundred kilobases," *Nature Methods*, vol. 6, no. 5, pp. 343–345, 2009.

[40] R. Silva-Rocha, E. Martínez-García, B. Calles, M. Chavarría, A. Arce-Rodríguez, A. De Las Heras, A. D. Páez-Espino, G. Durante-Rodríguez, J. Kim, P. I. Nikel, R. Platero, and V. De Lorenzo, "The Standard European Vector Architecture (SEVA): A coherent platform for the analysis and deployment of complex prokaryotic phenotypes," *Nucleic Acids Research*, vol. 41, no. D1, pp. 666–675, 2013.
